# *Gryon ancinla* Kozlov & Lê (Hymenoptera: Scelionidae): host association, expanded distribution, redescription and a new synonymy

**DOI:** 10.3897/BDJ.8.e47687

**Published:** 2020-10-26

**Authors:** Huayan Chen, Elijah J Talamas, Marie-Claude Bon, Matthew R. Moore

**Affiliations:** 1 State Key Laboratory of Biocontrol, School of Life Sciences, Sun Yat-sen University, Guangzhou, China State Key Laboratory of Biocontrol, School of Life Sciences, Sun Yat-sen University Guangzhou China; 2 Florida State Collection of Arthropods, Florida Department of Agriculture and Consumer Services, Gainesville, United States of America Florida State Collection of Arthropods, Florida Department of Agriculture and Consumer Services Gainesville United States of America; 3 Systematic Entomology Laboratory, United States Department of Agriculture, Washington, DC, United States of America Systematic Entomology Laboratory, United States Department of Agriculture Washington, DC United States of America; 4 USDA-ARS-EBCL, Montpellier, France USDA-ARS-EBCL Montpellier France; 5 Florida Department of Agriculture and Consumer Services, Gainesville, FL, United States of America Florida Department of Agriculture and Consumer Services Gainesville, FL United States of America

## Abstract

**Background:**

*Gryon* Haliday (Platygastroidea: Scelionidae) is a cosmopolitan genus of egg-parasitoid wasps primarily associated with Heteroptera.

**New information:**

*Gryon ancinla* Kozlov & Lê is reported for the first time outside of Vietnam, in China and Cambodia, and as an egg parasitoid of the pestiferous leaf-footed bug, *Acanthocoris scaber* (L.). *Gryon ancinla* is redescribed based on recently collected specimens and compared to closely related species of *Gryon* in the region. *Gryon clavaerus* Kozlov & Lê is treated as a junior synonym and some characters found in the *charon* species group are discussed.

## Introduction

**Figure 1.**
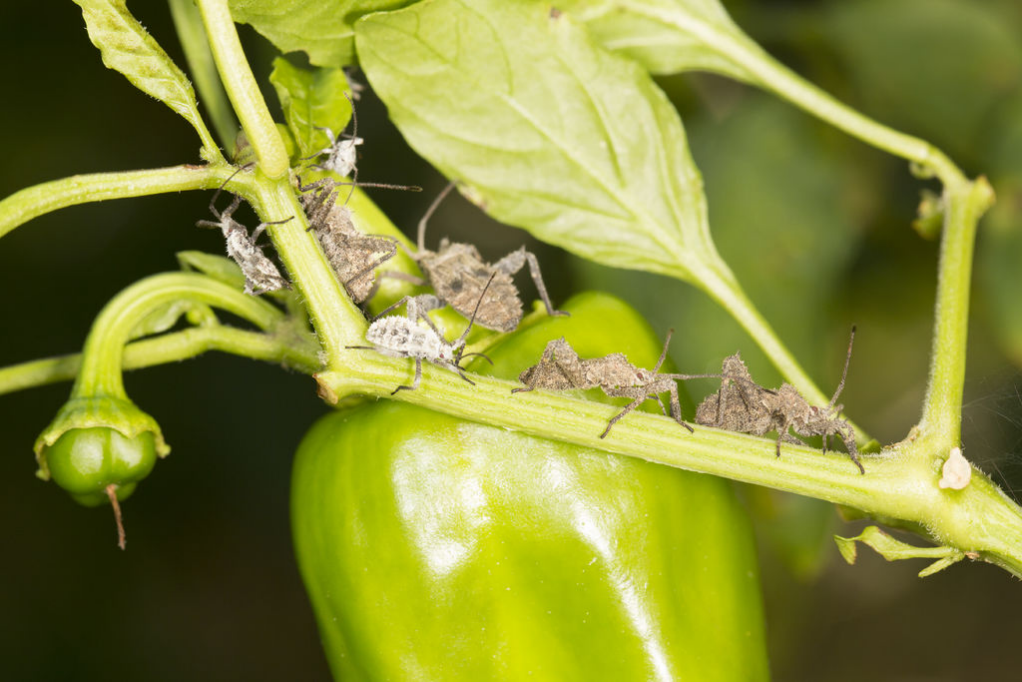
Nymphs and adults of *Acanthocoris scaber* feeding on a chili plant in Xiangtoushan, China.

*Acanthocoris scaber* (L.) (Hemiptera: Coreidae) is a pest that feeds on many economically important vegetables including chili, potato, tomato, and eggplant in China (Du et al. 2014), where it has become a serious problem for chili production in recent years. Adults and nymphs usually aggregate on leaves, stems and fruits (Fig. 1). They feed by sucking plant fluids that contain sugars and other nutrients, resulting in production losses. A recent survey conducted in China to assess parasitism of *A. scaber* eggs yielded numerous specimens of *Gryon* Haliday (Scelionidae), a cosmopolitan genus of parasitoid wasps whose species primarily parasitize the eggs of Heteroptera (Johnson et al. 1996, Masner 1983).

The taxonomy of *Gryon* in southeast Asia is in need of thorough revision, as in much of the world. The conspicuous characters of the *charon* species group, to which *G. ancinla* belongs, enabled us to consider a limited number of species names when identifying the specimens. We considered the possibility that some species may have ranges that extend from Asia into Africa. Indeed, *G. ancinla* is similar to some African *Gryon* species but the molecular data at hand indicate that it is distinct from the African species in the current analysis. We assign the name *G. ancinla* to these specimens because we are confident that their morphology matches that of the holotype specimen and they are from the same geographic region. There remain a number of Asian species in the *charon* species group for which we have not yet examined primary types, and examination of more material is required to fully assess the morphological variation and geographic range of *G. ancinla*. This study should thus be considered as one of many steps forward in the advancement of the taxonomy of *Gryon* and the reader should be aware that future work will undoubtedly result in more nomeclatural adjustments.

**Figure 4.**
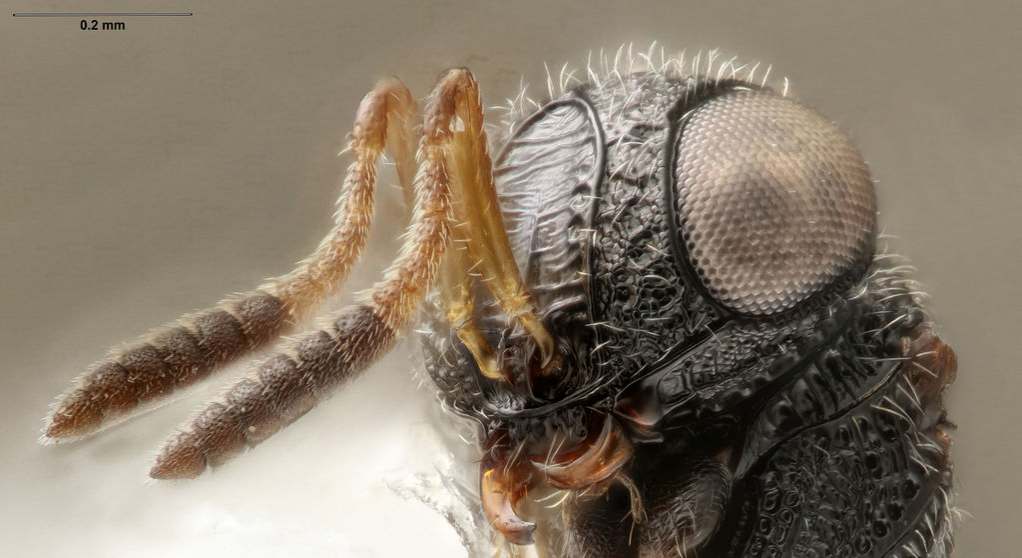
*Gryon ancinla*, female (FSCA 00090950), head and mesosoma, ventrolateral view.

The *charon* species group was proposed by Mineo 1983 for species with the frontal depression surrounded by a robust carina (Figs 2a, b, 4). Mineo also used other characters to define this group, many of which are located on the posterior head. While characters of the posterior head can be useful, their observation may require removal of the head from the mesosoma and it is not permissible to do with many primary type specimens. Mineo 1990 erected another species group, the *letus* group, listing only characters that this cluster shares with the *charon* group and did not provide characters to separate these groups. We consider this to be an unnecessary separation and, for simplicity, consider species with a frontal depression surrounded by a robust carina to be part of the *charon* group. A list of these species, while not exhaustive, is provided in Table 3 to facilitate meaningful comparisons between species. Phylogenetic analysis of *Gryon* is currently underway and we prefer not to further define any species groups without the context of a larger study. We also emphasize the use of characters that can be seen without dissection so that recently collected material can be matched to primary types. In *Gryon* there are many such characters to be exploited and during this study we found two that we have not previously encountered in taxonomic literature on the genus.

## Materials and methods

### Cybertaxonomy

The description of *G. ancinla* was generated using vSysLab (vsyslab.osu.edu), an online, matrix-based tool for generating species descriptions.

### Imaging

Specimens SCAU 3040175–3040176 were photographed using CombineZP and AutoMontage extended-focus software, using a JVC KY-F75U digital camera, Leica Z16 APOA microscope and 1X objective lens. Specimens were photographed using a Macroscopic Solutions Macropod using a 20X Mitutoyo objective lens with images rendered using Helicon Focus. Scanning electron micrographs were produced using Phenom Pro Desktop SEM and aligned in Helicon focus.

### Collections

Comprehensive identification tools are not presently available for *Gryon* in southeast Asia. Our determination of *G. ancinla* was made through direct comparison with holotypes and images of holotypes in the Institute of Ecology and Biological Resources (IEBR) (Hanoi, Vietnam) (Talamas and Pham 2017), The National Museum of Natural History (Washington, DC, USA) (Talamas et al. 2017), The Canadian National Collection of Insects (Ottawa, Canada), The Natural History Museum (NHMUK) (London, UK), Museo Civico di Storia Naturale Giacomo Doria (Genoa, Italy), National Museum of Natural History (Paris, France) and the Zoological Institute (St. Petersburg, Russia). The taxonomic and molecular portions of this study were based on material from the California Department of Food and Agriculture (CDFA), Florida State Collection of Arthropods, Gainesville, Florida (FSCA), Sun Yat-sen University, Guangzhou, China (SYSU), the Iziko Museum of Cape Town, South Africa (SAMC), the European Biological Control Laboratory, Montpellier, France (EBCL), and the College for Agriculture and Life Sciences, Seoul National University, Seoul, Korea (SNU).

### CO1 barcoding

Genomic DNA was nondestructively isolated from the entire specimen using a Qiagen DNeasy Blood and Tissue kit (Hilden, Germany). The barcode region of the mitochondrial Cytochrome c Oxidase Subunit I (CO1) was amplified using the universal barcoding primer sets LCO1490/HCO2198 (Folmer et al. 1994) or LEPF1/FEPR1 (Hebert et al. 2004). Amplification and sequence editing were done at EBCL as described in Sabbatini Peverieri et al. 2018. At FSCA, PCRs used the following thermocycle: 1) initial denaturation at 95^0^C for 2 minutes, 2) 98^0^C for 30 seconds, 3) 50^0^C for 30 seconds, 4) 72^0^C for 40 seconds [32x steps 2-4], and a final extension at 72^0^C for 7 minutes. Samples at FSCA were sequenced bidirectionally on the ABI SeqStudio platform with BigDye v3.1 chemistry. Sequences were trimmed and assembled into contigs using Sequencher 5.4.6.

All sequences generated from this study are deposited in GenBank (MT604053–MT604066) and all residual DNAs are archived at EBCL, FSCA, and SYSU (Table 2). Voucher specimens which have been reexamined following the molecular analysis were deposited in public collections (Table 2). All barcode sequences were translated into amino acids to check for stop codons. The sequences obtained were compared with sequences present in GenBank using the Basic Local Alignment Search Tool (http://www.ncbi.nlm.nih.gov/BLASTn) and aligned with the barcode sequences of *Gryon* species previously available. BOLD (Ratnasingham and Hebert 2007) was similarly datamined for *Gryon* CO1 barcodes and evaluated for barcode identification success. Similar sequences not identified to at least genus-level were not included in neighbor-joining analyses or K2P distance calculations. Tree search was performed in MEGA7 using the K2P model with a 95% per site data coverage cutoff (Kumar et al. 2016, Kimura 1980). Branch support, calculated with 10,000 boostrap replicates, and distance calculations used the same parameters.

CO1 barcodes were translated into amino acids and aligned using the default settings of ClustalW (Thompson et al. 1994) as implemented in MEGA7 (Kumar et al. 2016). The amino acid alignment was then back-translated into DNA sequences for neighbor-joining and distance analyses. The resulting alignment contains 682 bp positions and 98 terminal taxa. A *Psix* species was chosen as an outgroup for neighbor-joining analysis based on the phylogenetic topologies recovered by Taekul et al. 2014.

## Taxon treatments

### Gryon ancinla

Kozlov & Lê, 1996

2C2F30B5-EA11-5F2C-AFFD-F2CDBC48EC8C

Gryon ancinla Kozlov & Lê, 1996, Lê 1996: 11 (original description); Lê 2000: 98, 102 (description, keyed, type information).
Gryon clavaerus Kozlov & Lê **syn. n.**, 1996, Lê 1996: 12 (original description); Lê 2000: 99, 108 (description, keyed, type information).

#### Materials

- **Type status:** Holotype. **Occurrence:** recordedBy: Elijah Talamas; individualCount: 1; sex: female; lifeStage: adult; **Taxon:** scientificName: Gryon ancinla; kingdom: Animalia; phylum: Arthropoda; class: Inseca; order: Hymenoptera; family: Scelionidae; genus: Gryon; specificEpithet: ancinla; scientificNameAuthorship: Kozlov & Lê; **Location:** country: Vietnam; stateProvince: Bac Thai; county: Phu Luong; locality: Quan Chun Village; locationRemarks: label transliteration "Bac Thai [Province], Phu Luong [District], Quan Chun Village, 16.IV.1986, A. Sharkov [leg.] " . "Holotypus Gryon ancinla sp. n. 86"; **Identification:** identifiedBy: Kozlov & Lę; dateIdentified: 1996; **Event:** eventDate: 05/16/1986; **Record Level:** institutionCode: Zoological Institute, St. Petersburg, Russia (ZIN); basisOfRecord: PreservedSpecimen
- **Type status:** Holotype. **Occurrence:** catalogNumber: IEBR 0170; individualCount: 1; sex: female; lifeStage: adult; occurrenceID: urn:lsid:biosci.ohio-state.edu:osuc_occurrences:IEBR__0170; **Taxon:** scientificNameID: urn:lsid:biosci.ohio-state.edu:osuc_names:179785; scientificName: Gryon clavaerus; kingdom: Animalia; phylum: Arthropoda; class: Hexapoda; order: Hymenoptera; family: Scelionidae ; genus: Gryon; specificEpithet: clavaerus; **Location:** country: Vietnam; stateProvince: Hanoi; locality: Hanoi, Hanoi Prov., Vietnam; decimalLatitude: 21.0333; decimalLongitude: 105.85; georeferenceSources: GEOnet; **Identification:** identifiedBy: Kozlov, M. A. (Mikhail Alexeevich) & Lê, X. H. (Xuân Huê); dateIdentified: 1996; **Event:** samplingProtocol: none specified; eventDate: 05/04/1979; verbatimEventDate: May-04-1979; **Record Level:** modified: 2019-08-12T10:05:37Z; language: en; institutionCode: Institute of Ecology and Biological Resources, Hanoi, Vietnam (IEBR); collectionCode: Insects; basisOfRecord: PreservedSpecimen; source: http://hol.osu.edu/spmInfo.html?id=IEBR%200170
- **Type status:** Other material. **Occurrence:** catalogNumber: FSCA 00094673; recordedBy: Elijah Talamas; individualCount: 1; sex: male; lifeStage: adult; **Taxon:** scientificName: Gryon ancinla; kingdom: Animalia; phylum: Arthropoda; class: Inseca; order: Hymenoptera; family: Scelionidae; genus: Gryon; specificEpithet: ancinla; scientificNameAuthorship: Kozlov & Lê; **Location:** country: China; stateProvince: Guangdong; municipality: Guangzhou; locality: Huofoshan; locationRemarks: [CHINA: Guangdong Prov., Guangzhou, Huofoshan, MT in jungle 7-15.VI.2017 23°10'50"N 113°23'26"E, Chen & Talamas][PL197][DNAVoucher]; verbatimCoordinates: 23°10'50"N 113°23'26"E; decimalLatitude: 23.180556; decimalLongitude: 113.390556; georeferenceProtocol: label; **Identification:** identifiedBy: Elijah Talamas; **Event:** samplingProtocol: Malaise trap; eventDate: 2017-6-7/15; **Record Level:** institutionCode: Florida State Collection of Arthropods, Gainesville, Florida, USA (FSCA); basisOfRecord: PreservedSpecimen
- **Type status:** Other material. **Occurrence:** catalogNumber: FSCA 00094672; recordedBy: Elijah Talamas; individualCount: 1; sex: female; lifeStage: adult; **Taxon:** scientificName: Gryon ancinla; kingdom: Animalia; phylum: Arthropoda; class: Inseca; order: Hymenoptera; family: Scelionidae; genus: Gryon; specificEpithet: ancinla; scientificNameAuthorship: Kozlov & Lê; **Location:** country: China; stateProvince: Guangdong; municipality: Guangzhou; locality: Huofoshan; locationRemarks: [CHINA: Guangdong Prov., Guangzhou, Huofoshan, MT in jungle 7-15.VI.2017 23°10'50"N 113°23'26"E, Chen & Talamas][PL197][DNAVoucher]; verbatimCoordinates: 23°10'50"N 113°23'26"E; decimalLatitude: 23.180556; decimalLongitude: 113.390556; georeferenceProtocol: label; **Identification:** identifiedBy: Elijah Talamas; **Event:** samplingProtocol: Malaise trap; eventDate: 2017-6-7/15; **Record Level:** institutionCode: Florida State Collection of Arthropods, Gainesville, Florida, USA (FSCA); basisOfRecord: PreservedSpecimen
- **Type status:** Other material. **Occurrence:** catalogNumber: FSCA 00094671; recordedBy: Elijah Talamas; individualCount: 1; sex: male; lifeStage: adult; **Taxon:** scientificName: Gryon ancinla; kingdom: Animalia; phylum: Arthropoda; class: Inseca; order: Hymenoptera; family: Scelionidae; genus: Gryon; specificEpithet: ancinla; scientificNameAuthorship: Kozlov & Lê; **Location:** country: China; stateProvince: Guangdong; municipality: Guangzhou; locality: Huofoshan; locationRemarks: [CHINA: Guangdong Prov., Guangzhou, Huofoshan, MT in jungle 7-15.VI.2017 23°10'50"N 113°23'26"E, Chen & Talamas][PL197][DNAVoucher]; verbatimCoordinates: 23°10'50"N 113°23'26"E; decimalLatitude: 23.180556; decimalLongitude: 113.390556; georeferenceProtocol: label; **Identification:** identifiedBy: Elijah Talamas; **Event:** samplingProtocol: Malaise trap; eventDate: 2017-6-7/15; **Record Level:** institutionCode: Florida State Collection of Arthropods, Gainesville, Florida, USA (FSCA); basisOfRecord: PreservedSpecimen
- **Type status:** Other material. **Occurrence:** catalogNumber: FSCA 00094670; recordedBy: Elijah Talamas; individualCount: 1; sex: female; lifeStage: adult; **Taxon:** scientificName: Gryon ancinla; kingdom: Animalia; phylum: Arthropoda; class: Inseca; order: Hymenoptera; family: Scelionidae; genus: Gryon; specificEpithet: ancinla; scientificNameAuthorship: Kozlov & Lê; **Location:** country: China; stateProvince: Guangdong; municipality: Guangzhou; locality: Huofoshan; locationRemarks: [CHINA: Guangdong Prov., Guangzhou, Huofoshan, MT in jungle 7-15.VI.2017 23°10'50"N 113°23'26"E, Chen & Talamas][PL197][DNAVoucher]; verbatimCoordinates: 23°10'50"N 113°23'26"E; decimalLatitude: 23.180556; decimalLongitude: 113.390556; georeferenceProtocol: label; **Identification:** identifiedBy: Elijah Talamas; **Event:** samplingProtocol: Malaise trap; eventDate: 2017-6-7/15; **Record Level:** institutionCode: Florida State Collection of Arthropods, Gainesville, Florida, USA (FSCA); basisOfRecord: PreservedSpecimen
- **Type status:** Other material. **Occurrence:** catalogNumber: USNMENT01335640; recordedBy: Elijah Talamas; individualCount: 1; sex: female; lifeStage: adult; **Taxon:** scientificName: Gryon ancinla; kingdom: Animalia; phylum: Arthropoda; class: Inseca; order: Hymenoptera; family: Scelionidae; genus: Gryon; specificEpithet: ancinla; scientificNameAuthorship: Kozlov & Lê; **Location:** country: China; stateProvince: Guangdong; municipality: Huizhou; locality: Nan Kun Shan Forest Park; locationRemarks: [CHINA: Guangdong Prov., Huizhou, Nan Kun Shan Forest Park, MT in tea field beside stream & forest 8-14.VI.2017 23°38'8"N, 113°51'4"E Chen & Talamas][GRYON115][DNA Voucher]; verbatimCoordinates: 23°38'8"N 113°51'4"E; decimalLatitude: 23.635556; decimalLongitude: 113.851111; georeferenceProtocol: label; **Identification:** identifiedBy: Elijah Talamas; **Event:** samplingProtocol: Malaise trap; eventDate: 2017-6-8/14; **Record Level:** institutionCode: Florida State Collection of Arthropods, Gainesville, Florida, USA (FSCA); basisOfRecord: PreservedSpecimen
- **Type status:** Other material. **Occurrence:** catalogNumber: USNMENT01335659; recordedBy: Elijah Talamas; individualCount: 1; sex: female; lifeStage: adult; **Taxon:** scientificName: Gryon ancinla; kingdom: Animalia; phylum: Arthropoda; class: Inseca; order: Hymenoptera; family: Scelionidae; genus: Gryon; specificEpithet: ancinla; scientificNameAuthorship: Kozlov & Lê; **Location:** country: China; stateProvince: Guangdong; municipality: Huizhou; locality: Nan Kun Shan Forest Park; locationRemarks: [CHINA: Guangdong Prov., Huizhou, Nan Kun Shan Forest Park, YPT 9-10.VI.2017 trail along stream, 23°37'6"N 113°51'33"E, Chen & Talamas][GRYON118][DNA Voucher]; verbatimCoordinates: 23°37'6"N 113°51'33"E; decimalLatitude: 23.618333; decimalLongitude: 113.859167; georeferenceProtocol: label; **Identification:** identifiedBy: Elijah Talamas; **Event:** samplingProtocol: Yellow pan trap; eventDate: 2017-6-9/10; **Record Level:** institutionCode: Florida State Collection of Arthropods, Gainesville, Florida, USA (FSCA); basisOfRecord: PreservedSpecimen
- **Type status:** Other material. **Occurrence:** catalogNumber: FSCA 00090590; recordedBy: Elijah Talamas; individualCount: 1; sex: female; lifeStage: adult; **Taxon:** scientificName: Gryon ancinla; kingdom: Animalia; phylum: Arthropoda; class: Inseca; order: Hymenoptera; family: Scelionidae; genus: Gryon; specificEpithet: ancinla; scientificNameAuthorship: Kozlov & Lê; **Location:** country: Cambodia; stateProvince: Khum Ta Tey Leu; locality: Cardamom Mtns.; locationRemarks: [CAMBODIA: Khum Ta Tey Leu, Cardamom Mtns. MT 11°59'48.4"N 103°25'12.4"E 7-JUL-2010, Seunghwan Lee][PL077][DNA Voucher]; verbatimCoordinates: 11°59'48.4"N 103°25'12.4"E; decimalLatitude: 11.996778; decimalLongitude: 103.420111; georeferenceProtocol: label; **Identification:** identifiedBy: Elijah Talamas; **Event:** samplingProtocol: Malaise trap; eventDate: 07/07/2010; **Record Level:** institutionCode: College for Agriculture and Life Sciences, Seoul National University, Seoul, Korea (SNU); basisOfRecord: PreservedSpecimen
- **Type status:** Other material. **Occurrence:** catalogNumber: FSCA 00094699; recordedBy: Elijah Talamas; individualCount: 1; sex: female; lifeStage: adult; **Taxon:** scientificName: Gryon ancinla; kingdom: Animalia; phylum: Arthropoda; class: Inseca; order: Hymenoptera; family: Scelionidae; genus: Gryon; specificEpithet: ancinla; scientificNameAuthorship: Kozlov & Lê; **Location:** country: China; stateProvince: Zhejiang; locality: Tianmu Mountain (West); verbatimElevation: 930 m; locationRemarks: [CHINA: Zhejiang Province Tianmu Mountain (West), 930 m 30°22'10"N, 119°24'52"E SD Gaimari, M Hauser [12-15] ex. Malaise trap, 21-26.vi.2012][PL220][DNA Voucher]; verbatimCoordinates: 30°22'10"N 119°24'52"E; decimalLatitude: 30.369444; decimalLongitude: 119.414444; georeferenceProtocol: label; **Identification:** identifiedBy: Elijah Talamas; **Event:** samplingProtocol: Malaise trap; eventDate: 2012-6-21/26; **Record Level:** institutionCode: California Department of Food and Agriculture, Sacramento, California, USA (CDFA); basisOfRecord: PreservedSpecimen
- **Type status:** Other material. **Occurrence:** catalogNumber: FSCA 00094700; recordedBy: Elijah Talamas; individualCount: 1; sex: female; lifeStage: adult; **Taxon:** scientificName: Gryon ancinla; kingdom: Animalia; phylum: Arthropoda; class: Inseca; order: Hymenoptera; family: Scelionidae; genus: Gryon; specificEpithet: ancinla; scientificNameAuthorship: Kozlov & Lê; **Location:** country: China; stateProvince: Zhejiang; locality: Tianmu Mountain (West); verbatimElevation: 930 m; locationRemarks: [CHINA: Zhejiang Province Tianmu Mountain (West), 930m 30°22'10"N, 119°24'52"E SD Gaimari, M Hauser [12-15] ex. Malaise trap, 21-26.vi.2012][RA14][DNA Voucher]; verbatimCoordinates: 30°22'10"N 119°24'52"E; decimalLatitude: 30.369444; decimalLongitude: 119.414444; georeferenceProtocol: label; **Identification:** identifiedBy: Elijah Talamas; **Event:** samplingProtocol: Malaise trap; eventDate: 2012-6-21/26; **Record Level:** institutionCode: California Department of Food and Agriculture, Sacramento, California, USA (CDFA); basisOfRecord: PreservedSpecimen
- **Type status:** Other material. **Occurrence:** catalogNumber: SCAU 3040467; recordedBy: Chen, H. (Huayan); individualCount: 1; sex: female; lifeStage: adult; occurrenceID: urn:lsid:biosci.ohio-state.edu:osuc_occurrences:SCAU__3040467; **Taxon:** scientificNameID: urn:lsid:biosci.ohio-state.edu:osuc_names:4343; scientificName: Gryon ancinla; kingdom: Animalia; phylum: Arthropoda; class: Hexapoda; order: Hymenoptera; family: Scelionidae; genus: Gryon; specificEpithet: ancinla; **Location:** country: China; stateProvince: Guangdong; county: Huizhou; locality: Xiangtou Mountain, 23°15'50"N 114°22'16"E, Huizhou City, Guangdong Prov., China; decimalLatitude: 23.2639; decimalLongitude: 114.3711; georeferenceSources: label; **Identification:** identifiedBy: Talamas, E. J. (Elijah Jacob); dateIdentified: 2019; **Event:** samplingProtocol: reared from egg; eventDate: 08/21/2019; verbatimEventDate: Aug-21-2019; fieldNotes: [CHINA: Guangdong, Huizhou, Mt. Xiangtoushan, 23°15′50″N 114°22′16″E, ex. Eggs of Acanthocoris scaber, 21.viii.2019, Huayan Chen]; **Record Level:** modified: 2019-10-09T17:19:20Z; language: en; institutionCode: Sun Yat-sen University, Guangzhou, China (SYSU); collectionCode: Insects; basisOfRecord: PreservedSpecimen; source: http://hol.osu.edu/spmInfo.html?id=SCAU%203040467
- **Type status:** Other material. **Occurrence:** catalogNumber: SCAU 3017207; recordedBy: Chen, H. (Huayan); individualCount: 1; sex: male; lifeStage: adult; occurrenceID: urn:lsid:biosci.ohio-state.edu:osuc_occurrences:SCAU__3017207; **Taxon:** scientificNameID: urn:lsid:biosci.ohio-state.edu:osuc_names:4343; scientificName: Gryon ancinla; kingdom: Animalia; phylum: Arthropoda; class: Hexapoda; order: Hymenoptera; family: Scelionidae; genus: Gryon; specificEpithet: ancinla; **Location:** country: China; stateProvince: Guangdong; county: Guangzhou; locality: Guangzhou University Town, 23°03'21"N 113°24'41"E, Guangzhou City, Guangdong Prov., China; decimalLatitude: 23.0558; decimalLongitude: 113.4114; georeferenceSources: label; **Identification:** identifiedBy: Talamas, E. J. (Elijah Jacob); dateIdentified: 2019; **Event:** samplingProtocol: reared from egg; eventDate: 06/18/2019; verbatimEventDate: Jun-18-2019; fieldNotes: [CHINA: Guangdong, Guangzhou, University Town, 23°3′21″N 113°24′41″E, ex. Eggs of Acanthocoris scaber, 18.vi.2019, Huayan Chen]; **Record Level:** modified: 2019-10-09T17:37:10Z; language: en; institutionCode: Sun Yat-sen University, Guangzhou, China (SYSU); collectionCode: Insects; basisOfRecord: PreservedSpecimen; source: http://hol.osu.edu/spmInfo.html?id=SCAU%203017207
- **Type status:** Other material. **Occurrence:** catalogNumber: SCAU 3040175; recordedBy: Chen, H. (Huayan); individualCount: 1; sex: female; lifeStage: adult; occurrenceID: urn:lsid:biosci.ohio-state.edu:osuc_occurrences:SCAU__3040175; **Taxon:** scientificNameID: urn:lsid:biosci.ohio-state.edu:osuc_names:4343; scientificName: Gryon ancinla; kingdom: Animalia; phylum: Arthropoda; class: Hexapoda; order: Hymenoptera; family: Scelionidae; genus: Gryon; specificEpithet: ancinla; **Location:** country: China; stateProvince: Guangdong; county: Guangzhou; locality: Guangzhou University Town, 23°03'21"N 113°24'41"E, Guangzhou City, Guangdong Prov., China; decimalLatitude: 23.0558; decimalLongitude: 113.4114; georeferenceSources: label; **Identification:** identifiedBy: Talamas, E. J. (Elijah Jacob); dateIdentified: 2019; **Event:** samplingProtocol: reared from egg; eventDate: 06/18/2019; verbatimEventDate: Jun-18-2019; fieldNotes: [CHINA: Guangdong, Guangzhou, University Town, 23°3′21″N 113°24′41″E, ex. Eggs of Acanthocoris scaber, 18.vi.2019, Huayan Chen]; **Record Level:** modified: 2019-10-09T17:40:42Z; language: en; institutionCode: Sun Yat-sen University, Guangzhou, China (SYSU); collectionCode: Insects; basisOfRecord: PreservedSpecimen; source: http://hol.osu.edu/spmInfo.html?id=SCAU%203040175
- **Type status:** Other material. **Occurrence:** catalogNumber: SCAU 3040470; recordedBy: Chen, H. (Huayan); individualCount: 1; sex: female; lifeStage: adult; occurrenceID: urn:lsid:biosci.ohio-state.edu:osuc_occurrences:SCAU__3040470; **Taxon:** scientificNameID: urn:lsid:biosci.ohio-state.edu:osuc_names:4343; scientificName: Gryon ancinla; kingdom: Animalia; phylum: Arthropoda; class: Hexapoda; order: Hymenoptera; family: Scelionidae; genus: Gryon; specificEpithet: ancinla; **Location:** country: China; stateProvince: Guangdong; county: Huizhou; locality: Xiangtou Mountain, 23°15'50"N 114°22'16"E, Huizhou City, Guangdong Prov., China; decimalLatitude: 23.2639; decimalLongitude: 114.3711; georeferenceSources: label; **Identification:** identifiedBy: Talamas, E. J. (Elijah Jacob); dateIdentified: 2019; **Event:** samplingProtocol: reared from egg; eventDate: 08/21/2019; verbatimEventDate: Aug-21-2019; fieldNotes: [CHINA: Guangdong, Huizhou, Mt. Xiangtoushan, 23°15′50″N 114°22′16″E, ex. Eggs of Acanthocoris scaber, 21.viii.2019, Huayan Chen]; **Record Level:** modified: 2019-10-09T17:30:00Z; language: en; institutionCode: Sun Yat-sen University, Guangzhou, China (SYSU); collectionCode: Insects; basisOfRecord: PreservedSpecimen; source: http://hol.osu.edu/spmInfo.html?id=SCAU%203040470
- **Type status:** Other material. **Occurrence:** catalogNumber: SCAU 3040987; recordedBy: Chen, H. (Huayan); individualCount: 1; sex: female; lifeStage: adult; occurrenceID: urn:lsid:biosci.ohio-state.edu:osuc_occurrences:SCAU__3040987; **Taxon:** scientificNameID: urn:lsid:biosci.ohio-state.edu:osuc_names:4343; scientificName: Gryon ancinla; kingdom: Animalia; phylum: Arthropoda; class: Hexapoda; order: Hymenoptera; family: Scelionidae; genus: Gryon; specificEpithet: ancinla; **Location:** country: China; stateProvince: Guangdong; county: Guangzhou; locality: Guangzhou University Town, 23°03'21"N 113°24'41"E, Guangzhou City, Guangdong Prov., China; decimalLatitude: 23.0558; decimalLongitude: 113.4114; georeferenceSources: label; **Identification:** identifiedBy: Talamas, E. J. (Elijah Jacob); dateIdentified: 2019; **Event:** samplingProtocol: reared from egg; eventDate: 06/18/2019; verbatimEventDate: Jun-18-2019; fieldNotes: [CHINA: Guangdong, Guangzhou, University Town, 23°3′21″N 113°24′41″E, ex. Eggs of Acanthocoris scaber, 18.vi.2019, Huayan Chen]; **Record Level:** modified: 2019-10-09T17:54:54Z; language: en; institutionCode: Sun Yat-sen University, Guangzhou, China (SYSU); collectionCode: Insects; basisOfRecord: PreservedSpecimen; source: http://hol.osu.edu/spmInfo.html?id=SCAU%203040987
- **Type status:** Other material. **Occurrence:** catalogNumber: SCAU 3040988; recordedBy: Chen, H. (Huayan); individualCount: 1; sex: female; lifeStage: adult; occurrenceID: urn:lsid:biosci.ohio-state.edu:osuc_occurrences:SCAU__3040988; **Taxon:** scientificNameID: urn:lsid:biosci.ohio-state.edu:osuc_names:4343; scientificName: Gryon ancinla; kingdom: Animalia; phylum: Arthropoda; class: Hexapoda; order: Hymenoptera; family: Scelionidae; genus: Gryon; specificEpithet: ancinla; **Location:** country: China; stateProvince: Guangdong; county: Guangzhou; locality: Guangzhou University Town, 23°03'21"N 113°24'41"E, Guangzhou City, Guangdong Prov., China; decimalLatitude: 23.0558; decimalLongitude: 113.4114; georeferenceSources: label; **Identification:** identifiedBy: Talamas, E. J. (Elijah Jacob); dateIdentified: 2019; **Event:** samplingProtocol: reared from egg; eventDate: 06/18/2019; verbatimEventDate: Jun-18-2019; fieldNotes: [CHINA: Guangdong, Guangzhou, University Town, 23°3′21″N 113°24′41″E, ex. Eggs of Acanthocoris scaber, 18.vi.2019, Huayan Chen]; **Record Level:** modified: 2019-10-09T17:56:56Z; language: en; institutionCode: Sun Yat-sen University, Guangzhou, China (SYSU); collectionCode: Insects; basisOfRecord: PreservedSpecimen; source: http://hol.osu.edu/spmInfo.html?id=SCAU%203040988
- **Type status:** Other material. **Occurrence:** catalogNumber: SCAU 3040990; recordedBy: Chen, H. (Huayan); individualCount: 1; sex: female; lifeStage: adult; occurrenceID: urn:lsid:biosci.ohio-state.edu:osuc_occurrences:SCAU__3040990; **Taxon:** scientificNameID: urn:lsid:biosci.ohio-state.edu:osuc_names:4343; scientificName: Gryon ancinla; kingdom: Animalia; phylum: Arthropoda; class: Hexapoda; order: Hymenoptera; family: Scelionidae; genus: Gryon; specificEpithet: ancinla; **Location:** country: China; stateProvince: Guangdong; county: Guangzhou; locality: Guangzhou University Town, 23°03'21"N 113°24'41"E, Guangzhou City, Guangdong Prov., China; decimalLatitude: 23.0558; decimalLongitude: 113.4114; georeferenceSources: label; **Identification:** identifiedBy: Talamas, E. J. (Elijah Jacob); dateIdentified: 2019; **Event:** samplingProtocol: reared from egg; eventDate: 06/18/2019; verbatimEventDate: Jun-18-2019; fieldNotes: [CHINA: Guangdong, Guangzhou, University Town, 23°3′21″N 113°24′41″E, ex. Eggs of Acanthocoris scaber, 18.vi.2019, Huayan Chen]; **Record Level:** modified: 2019-10-09T18:05:14Z; language: en; institutionCode: Sun Yat-sen University, Guangzhou, China (SYSU); collectionCode: Insects; basisOfRecord: PreservedSpecimen; source: http://hol.osu.edu/spmInfo.html?id=SCAU%203040990
- **Type status:** Other material. **Occurrence:** catalogNumber: SCAU 3040989; recordedBy: Chen, H. (Huayan); individualCount: 1; sex: female; lifeStage: adult; occurrenceID: urn:lsid:biosci.ohio-state.edu:osuc_occurrences:SCAU__3040989; **Taxon:** scientificNameID: urn:lsid:biosci.ohio-state.edu:osuc_names:4343; scientificName: Gryon ancinla; kingdom: Animalia; phylum: Arthropoda; class: Hexapoda; order: Hymenoptera; family: Scelionidae; genus: Gryon; specificEpithet: ancinla; **Location:** country: China; stateProvince: Guangdong; county: Guangzhou; locality: Guangzhou University Town, 23°03'21"N 113°24'41"E, Guangzhou City, Guangdong Prov., China; decimalLatitude: 23.0558; decimalLongitude: 113.4114; georeferenceSources: label; **Identification:** identifiedBy: Talamas, E. J. (Elijah Jacob); dateIdentified: 2019; **Event:** samplingProtocol: reared from egg; eventDate: 06/18/2019; verbatimEventDate: Jun-18-2019; fieldNotes: [CHINA: Guangdong, Guangzhou, University Town, 23°3′21″N 113°24′41″E, ex. Eggs of Acanthocoris scaber, 18.vi.2019, Huayan Chen]; **Record Level:** modified: 2019-10-09T18:08:09Z; language: en; institutionCode: Sun Yat-sen University, Guangzhou, China (SYSU); collectionCode: Insects; basisOfRecord: PreservedSpecimen; source: http://hol.osu.edu/spmInfo.html?id=SCAU%203040989
- **Type status:** Other material. **Occurrence:** catalogNumber: SCAU 3040992; recordedBy: Chen, H. (Huayan); individualCount: 1; sex: female; lifeStage: adult; occurrenceID: urn:lsid:biosci.ohio-state.edu:osuc_occurrences:SCAU__3040992; **Taxon:** scientificNameID: urn:lsid:biosci.ohio-state.edu:osuc_names:4343; scientificName: Gryon ancinla; kingdom: Animalia; phylum: Arthropoda; class: Hexapoda; order: Hymenoptera; family: Scelionidae; genus: Gryon; specificEpithet: ancinla; **Location:** country: China; stateProvince: Guangdong; county: Guangzhou; locality: Guangzhou University Town, 23°03'21"N 113°24'41"E, Guangzhou City, Guangdong Prov., China; decimalLatitude: 23.0558; decimalLongitude: 113.4114; georeferenceSources: label; **Identification:** identifiedBy: Talamas, E. J. (Elijah Jacob); dateIdentified: 2019; **Event:** samplingProtocol: reared from egg; eventDate: 06/18/2019; verbatimEventDate: Jun-18-2019; fieldNotes: [CHINA: Guangdong, Guangzhou, University Town, 23°3′21″N 113°24′41″E, ex. Eggs of Acanthocoris scaber, 18.vi.2019, Huayan Chen]; **Record Level:** modified: 2019-10-09T18:11:10Z; language: en; institutionCode: Sun Yat-sen University, Guangzhou, China (SYSU); collectionCode: Insects; basisOfRecord: PreservedSpecimen; source: http://hol.osu.edu/spmInfo.html?id=SCAU%203040992
- **Type status:** Other material. **Occurrence:** catalogNumber: SCAU 3040993; recordedBy: Chen, H. (Huayan); individualCount: 1; sex: female; lifeStage: adult; occurrenceID: urn:lsid:biosci.ohio-state.edu:osuc_occurrences:SCAU__3040993; **Taxon:** scientificNameID: urn:lsid:biosci.ohio-state.edu:osuc_names:4343; scientificName: Gryon ancinla; kingdom: Animalia; phylum: Arthropoda; class: Hexapoda; order: Hymenoptera; family: Scelionidae; genus: Gryon; specificEpithet: ancinla; **Location:** country: China; stateProvince: Guangdong; county: Guangzhou; locality: Guangzhou University Town, 23°03'21"N 113°24'41"E, Guangzhou City, Guangdong Prov., China; decimalLatitude: 23.0558; decimalLongitude: 113.4114; georeferenceSources: label; **Identification:** identifiedBy: Talamas, E. J. (Elijah Jacob); dateIdentified: 2019; **Event:** samplingProtocol: reared from egg; eventDate: 06/18/2019; verbatimEventDate: Jun-18-2019; fieldNotes: [CHINA: Guangdong, Guangzhou, University Town, 23°3′21″N 113°24′41″E, ex. Eggs of Acanthocoris scaber, 18.vi.2019, Huayan Chen]; **Record Level:** modified: 2019-10-09T18:14:04Z; language: en; institutionCode: Sun Yat-sen University, Guangzhou, China (SYSU); collectionCode: Insects; basisOfRecord: PreservedSpecimen; source: http://hol.osu.edu/spmInfo.html?id=SCAU%203040993
- **Type status:** Other material. **Occurrence:** catalogNumber: SCAU 3040469; recordedBy: Chen, H. (Huayan); individualCount: 1; sex: female; lifeStage: adult; occurrenceID: urn:lsid:biosci.ohio-state.edu:osuc_occurrences:SCAU__3040469; **Taxon:** scientificNameID: urn:lsid:biosci.ohio-state.edu:osuc_names:4343; scientificName: Gryon ancinla; kingdom: Animalia; phylum: Arthropoda; class: Hexapoda; order: Hymenoptera; family: Scelionidae; genus: Gryon; specificEpithet: ancinla; **Location:** country: China; stateProvince: Guangdong; county: Huizhou; locality: Xiangtou Mountain, 23°15'50"N 114°22'16"E, Huizhou City, Guangdong Prov., China; decimalLatitude: 23.2639; decimalLongitude: 114.3711; georeferenceSources: label; **Identification:** identifiedBy: Talamas, E. J. (Elijah Jacob); dateIdentified: 2019; **Event:** samplingProtocol: reared from egg; eventDate: 08/21/2019; verbatimEventDate: Aug-21-2019; fieldNotes: [CHINA: Guangdong, Huizhou, Mt. Xiangtoushan, 23°15′50″N 114°22′16″E, ex. Eggs of Acanthocoris scaber, 21.viii.2019, Huayan Chen]; **Record Level:** modified: 2019-10-09T17:28:15Z; language: en; institutionCode: Sun Yat-sen University, Guangzhou, China (SYSU); collectionCode: Insects; basisOfRecord: PreservedSpecimen; source: http://hol.osu.edu/spmInfo.html?id=SCAU%203040469
- **Type status:** Other material. **Occurrence:** catalogNumber: SCAU 3040985; recordedBy: Chen, H. (Huayan); individualCount: 1; sex: female; lifeStage: adult; occurrenceID: urn:lsid:biosci.ohio-state.edu:osuc_occurrences:SCAU__3040985; **Taxon:** scientificNameID: urn:lsid:biosci.ohio-state.edu:osuc_names:4343; scientificName: Gryon ancinla; kingdom: Animalia; phylum: Arthropoda; class: Hexapoda; order: Hymenoptera; family: Scelionidae; genus: Gryon; specificEpithet: ancinla; **Location:** country: China; stateProvince: Guangdong; county: Guangzhou; locality: Guangzhou University Town, 23°03'21"N 113°24'41"E, Guangzhou City, Guangdong Prov., China; decimalLatitude: 23.0558; decimalLongitude: 113.4114; georeferenceSources: label; **Identification:** identifiedBy: Talamas, E. J. (Elijah Jacob); dateIdentified: 2019; **Event:** samplingProtocol: reared from egg; eventDate: 06/18/2019; verbatimEventDate: Jun-18-2019; fieldNotes: [CHINA: Guangdong, Guangzhou, University Town, 23°3′21″N 113°24′41″E, ex. Eggs of Acanthocoris scaber, 18.vi.2019, Huayan Chen]; **Record Level:** modified: 2019-10-09T17:46:54Z; language: en; institutionCode: Sun Yat-sen University, Guangzhou, China (SYSU); collectionCode: Insects; basisOfRecord: PreservedSpecimen; source: http://hol.osu.edu/spmInfo.html?id=SCAU%203040985
- **Type status:** Other material. **Occurrence:** catalogNumber: SCAU 3040986; recordedBy: Chen, H. (Huayan); individualCount: 1; sex: female; lifeStage: adult; occurrenceID: urn:lsid:biosci.ohio-state.edu:osuc_occurrences:SCAU__3040986; **Taxon:** scientificNameID: urn:lsid:biosci.ohio-state.edu:osuc_names:4343; scientificName: Gryon ancinla; kingdom: Animalia; phylum: Arthropoda; class: Hexapoda; order: Hymenoptera; family: Scelionidae; genus: Gryon; specificEpithet: ancinla; **Location:** country: China; stateProvince: Guangdong; county: Guangzhou; locality: Guangzhou University Town, 23°03'21"N 113°24'41"E, Guangzhou City, Guangdong Prov., China; decimalLatitude: 23.0558; decimalLongitude: 113.4114; georeferenceSources: label; **Identification:** identifiedBy: Talamas, E. J. (Elijah Jacob); dateIdentified: 2019; **Event:** samplingProtocol: reared from egg; eventDate: 06/18/2019; verbatimEventDate: Jun-18-2019; fieldNotes: [CHINA: Guangdong, Guangzhou, University Town, 23°3′21″N 113°24′41″E, ex. Eggs of Acanthocoris scaber, 18.vi.2019, Huayan Chen]; **Record Level:** modified: 2019-10-09T17:48:47Z; language: en; institutionCode: Sun Yat-sen University, Guangzhou, China (SYSU); collectionCode: Insects; basisOfRecord: PreservedSpecimen; source: http://hol.osu.edu/spmInfo.html?id=SCAU%203040986
- **Type status:** Other material. **Occurrence:** catalogNumber: SCAU 3040468; recordedBy: Chen, H. (Huayan); individualCount: 1; sex: female; lifeStage: adult; occurrenceID: urn:lsid:biosci.ohio-state.edu:osuc_occurrences:SCAU__3040468; **Taxon:** scientificNameID: urn:lsid:biosci.ohio-state.edu:osuc_names:4343; scientificName: Gryon ancinla; kingdom: Animalia; phylum: Arthropoda; class: Hexapoda; order: Hymenoptera; family: Scelionidae; genus: Gryon; specificEpithet: ancinla; **Location:** country: China; stateProvince: Guangdong; county: Huizhou; locality: Xiangtou Mountain, 23°15'50"N 114°22'16"E, Huizhou City, Guangdong Prov., China; decimalLatitude: 23.2639; decimalLongitude: 114.3711; georeferenceSources: label; **Identification:** identifiedBy: Talamas, E. J. (Elijah Jacob); dateIdentified: 2019; **Event:** samplingProtocol: reared from egg; eventDate: 08/21/2019; verbatimEventDate: Aug-21-2019; fieldNotes: [CHINA: Guangdong, Huizhou, Mt. Xiangtoushan, 23°15′50″N 114°22′16″E, ex. Eggs of Acanthocoris scaber, 21.viii.2019, Huayan Chen]; **Record Level:** modified: 2019-10-09T17:19:42Z; language: en; institutionCode: Sun Yat-sen University, Guangzhou, China (SYSU); collectionCode: Insects; basisOfRecord: PreservedSpecimen; source: http://hol.osu.edu/spmInfo.html?id=SCAU%203040468
- **Type status:** Other material. **Occurrence:** catalogNumber: SCAU 3017206; recordedBy: Chen, H. (Huayan); individualCount: 1; sex: female; lifeStage: adult; occurrenceID: urn:lsid:biosci.ohio-state.edu:osuc_occurrences:SCAU__3017206; **Taxon:** scientificNameID: urn:lsid:biosci.ohio-state.edu:osuc_names:4343; scientificName: Gryon ancinla; kingdom: Animalia; phylum: Arthropoda; class: Hexapoda; order: Hymenoptera; family: Scelionidae; genus: Gryon; specificEpithet: ancinla; **Location:** country: China; stateProvince: Guangdong; county: Guangzhou; locality: Guangzhou University Town, 23°03'21"N 113°24'41"E, Guangzhou City, Guangdong Prov., China; decimalLatitude: 23.0558; decimalLongitude: 113.4114; georeferenceSources: label; **Identification:** identifiedBy: Talamas, E. J. (Elijah Jacob); dateIdentified: 2019; **Event:** samplingProtocol: reared from egg; eventDate: 06/18/2019; verbatimEventDate: Jun-18-2019; fieldNotes: [CHINA: Guangdong, Guangzhou, University Town, 23°3′21″N 113°24′41″E, ex. Eggs of Acanthocoris scaber, 18.vi.2019, Huayan Chen]; **Record Level:** modified: 2019-10-09T17:32:59Z; language: en; institutionCode: Sun Yat-sen University, Guangzhou, China (SYSU); collectionCode: Insects; basisOfRecord: PreservedSpecimen; source: http://hol.osu.edu/spmInfo.html?id=SCAU%203017206
- **Type status:** Other material. **Occurrence:** catalogNumber: SCAU 3040176; recordedBy: Chen, H. (Huayan); individualCount: 1; sex: male; lifeStage: adult; occurrenceID: urn:lsid:biosci.ohio-state.edu:osuc_occurrences:SCAU__3040176; **Taxon:** scientificNameID: urn:lsid:biosci.ohio-state.edu:osuc_names:4343; scientificName: Gryon ancinla; kingdom: Animalia; phylum: Arthropoda; class: Hexapoda; order: Hymenoptera; family: Scelionidae; genus: Gryon; specificEpithet: ancinla; **Location:** country: China; stateProvince: Guangdong; county: Guangzhou; locality: Guangzhou University Town, 23°03'21"N 113°24'41"E, Guangzhou City, Guangdong Prov., China; decimalLatitude: 23.0558; decimalLongitude: 113.4114; georeferenceSources: label; **Identification:** identifiedBy: Talamas, E. J. (Elijah Jacob); dateIdentified: 2019; **Event:** samplingProtocol: reared from egg; eventDate: 06/18/2019; verbatimEventDate: Jun-18-2019; fieldNotes: [CHINA: Guangdong, Guangzhou, University Town, 23°3′21″N 113°24′41″E, ex. Eggs of Acanthocoris scaber, 18.vi.2019, Huayan Chen]; **Record Level:** modified: 2019-10-09T17:44:43Z; language: en; institutionCode: Sun Yat-sen University, Guangzhou, China (SYSU); collectionCode: Insects; basisOfRecord: PreservedSpecimen; source: http://hol.osu.edu/spmInfo.html?id=SCAU%203040176
- **Type status:** Other material. **Occurrence:** catalogNumber: SCAU 3040994; recordedBy: Chen, H. (Huayan); individualCount: 1; sex: female; lifeStage: adult; occurrenceID: urn:lsid:biosci.ohio-state.edu:osuc_occurrences:SCAU__3040994; **Taxon:** scientificNameID: urn:lsid:biosci.ohio-state.edu:osuc_names:4343; scientificName: Gryon ancinla; kingdom: Animalia; phylum: Arthropoda; class: Hexapoda; order: Hymenoptera; family: Scelionidae; genus: Gryon; specificEpithet: ancinla; **Location:** country: China; stateProvince: Guangdong; county: Guangzhou; locality: Guangzhou University Town, 23°03'21"N 113°24'41"E, Guangzhou City, Guangdong Prov., China; decimalLatitude: 23.0558; decimalLongitude: 113.4114; georeferenceSources: label; **Identification:** identifiedBy: Talamas, E. J. (Elijah Jacob); dateIdentified: 2019; **Event:** samplingProtocol: reared from egg; eventDate: 06/18/2019; verbatimEventDate: Jun-18-2019; fieldNotes: [CHINA: Guangdong, Guangzhou, University Town, 23°3′21″N 113°24′41″E, ex. Eggs of Acanthocoris scaber, 18.vi.2019, Huayan Chen]; **Record Level:** modified: 2019-10-09T18:17:48Z; language: en; institutionCode: Sun Yat-sen University, Guangzhou, China (SYSU); collectionCode: Insects; basisOfRecord: PreservedSpecimen; source: http://hol.osu.edu/spmInfo.html?id=SCAU%203040994

#### Description

**Size**: Female body length: 1.26–1.86 mm (n=9).

**Color**: Color of body: dark brown to black, rarely with reddish-brown areas. Color of legs: coxae dark brown to black, otherwise yellow.

**Head**: Number of papillary sensilla on A7: 2. Number of papillary sensilla on A8: 2. Color of antenna in female: A1–A6 yellow, A7–A12 brown. Number of mandibular teeth: 3. Shape of mandibular teeth: dorsal tooth distinctly the largest. Shape of clypeus: roughly rectangular with rounded corners. Number of clypeal setae: 6. Epiclypeal carina: present. Facial striae: absent. Central keel: present. Line of setae above interantennal process: absent. Setation of compound eye: short and sparse, often appearing absent with light microscopy. Setation of orbital furrow: present along dorsal half of compound eye. Macrosculpture of frontal depression: transversely rugose. Sculpture of frons outside of frontal depression: areolate rugose. Lateral margin of frontal depression: delimited by carina. Dorsal margin of frontal depression: delimited by carina. Smooth area at base of mandible: present. Malar striae: absent. Genal carina: present. Hyperoccipital carina: present between lateral ocelli. Anterior margin of occipital carina on gena: crenulate. Occipital carina: terminating near dorsal margin of compound eye.

**Mesosoma**: Epomial carina: present. Sculpture of lateral pronotum: transversely rugose. Netrion sulcus: absent. Mesoscutal suprahumeral sulcus: absent. Mesoscutal humeral sulcus: present as a smooth furrow, anteriorly terminating in a pit. Sculpture of mesoscutum: coarsely rugose, with rugae oriented longitudinally at posterior margin. Posterior mesoscutellar sulcus: foveate. Posterior margin of mesoscutellum: extending over metanotum, metascutellum not visible in dorsal view. Posterior margin of metascutellum: straight. Sculpture on posteroventral surface metascutellum: antero-posteriorly strigose. Sculpture of metanotal trough: foveate. Length of postmarginal vein in fore wing: about twice as long as stigmal vein. Length of marginal vein in fore wing: about half as long as stigmal vein. Lateral propodeal carina: continuous across posterior propodeum, forming flange around metasomal depression. Sculpture of metasomal depression: radially strigose. Preacetabular sulcus: present as a line of punctures. Orientation of acetabular carina: parallel to mesopleural carina. Posterior limit of acetabulum: nearly reaching ventral mesopleural carina. Postacetabular sulcus: crenulate. Episternal foveae: absent. Mesopleural carina: present. Cells or foveae along ventral margin of mesopleural carina: present. Sculpture of femoral depression: irregularly rugose. Prespecular sulcus: indicated by crenulae. Sculpture of speculum: transversely rugose. Mesepimeral sulcus: comprised of circular foveae. Sculpture of posterior mesepimeral area: weakly rugulose in ventral half. Paracoxal sulcus: indicated by large, irregular cells along anterior margin of metapleuron. Anteroventral extension of the metapleuron: long, reaching base of mesocoxa. Metapleural structure: dorsoventrally divided by carina, posterior portion densely setose.

**Metasoma**: Form of sulcus on anterior T1: simple line of foveae. Lateral pit on anterior T1: absent. Macrosculpture of T1: longitudinally striate. Setation of T1: present in a posterolateral triangular area. Smooth area on anterior T2: present. Setation of T2: sparse medially, dense laterally. Macrosculpture of T2: irregularly rugose. Posterior margin of T6: concave. Lateral pit on anterior S1: absent. Transverse sulcus on anterior S2: absent. Macrosculpture of S2: sparsely striate, striae attenuating posteriorly.

**Variation**: Specimens FSCA 00094670 and FSCA 00094672 (Fig. 10) are both female, are from the same Malaise trap sample and have identical CO1 barcode sequences. They are also notably different in size (1.67 and 1.27 mm, respectively) and exhibit differences in the sculpture of the frons between the frontal depression and the inner orbit of the compound eye. Specimen FSCA 00094670, which is the larger specimen, has a ridge extending from the orbital carina to the margin of the frontal depression (Fig. 10a). Interestingly, the location of this ridge along the inner orbit corresponds to the transition point between the setose and glabrous portions of the orbital furrow. This ridge can clearly be seen in the holotype specimen of *G. ancinla* (Fig. 2b). The smaller of the two specimens, FSCA 00094672, has the frons evenly rugose between the orbital furrow and the frontal depression, without a transverse ridge (Fig. 10b).

#### Diagnosis

Females of *G. ancinla* have a 6-merous clava (Fig. 4), which is found in other species of the *charon* group, including the African species *G. charon* and *G. paracharontis*. The holotype of *G. sponus*, from Vietnam, is missing its antennae (Fig. 5), but the illustration of the female antenna in Lê 1996 suggests that it has 6 clavomeres. Each of these species can also be separated from *G. ancinla* by the shape of the mesoscutellum. In *G. ancinla*, the posterior margin of the mesoscutellar disc is directly above the posterior margin of the scutellar rim. In *G. charon*, *G. paracharontis*, and *G. sponus*, the mesoscutellar disc extends posteriorly well beyond the scutellar rim (Fig. 5). *Gryon drunoris*, which is sympatric with *G. ancinla*, has a mesoscutellum that is evenly convex and the clava is 5-merous (Fig. 6). *Gryon ancinla* and *G. drunoris* may also be separated by the relative lengths of the metasomal tergites: T1 is distinctly longer than T3 in *G. ancinla* (Fig. 7a) and they are roughly equal in *G. drunoris* (Fig. 7b). In the females of the *charon* species group that we have examined so far, the clava tends to be distinctly darker than the preceding antennomeres. This makes the clava easily distinguishable from the funicle in most cases, but we caution that using color to differentiate the clava from the funicle may not be reliable in all species or specimens and unambiguous determination of the number of clavomeres requires examination of the papillary sensilla.

#### Notes

*Gryon clavaerus* (Fig. 3) was described in the same publication as *G. ancinla*. Our examination of the type specimens finds no differences that justify keeping them as separate species and we thus treat *G. clavaerus* as a junior synonym.

## Analysis

### Characters in the *charon* species group

**Humeral pit**

The mesoscutal humeral pit (Fig. 8) is located at the junction of the mesoscutal humeral sulcus and the mesoscutal suprahumeral sulcus. This pit is found in all species of the *charon* group that we have examined. It is also present in species that are not part of the *charon* group as it is currently defined, and these species have varying forms of carinae surrounding the frontal depression. The mesoscutal humeral pit thus may be useful for determining affinities between the *charon* group and other lineages within *Gryon*.

**Setation of the orbital furrow**

Setation of the orbital furrow can separate some species in the *charon* group and perhaps other species groups. *Gryon ancinla* has setation only in the dorsal part of the orbital furrow (Figs 2b, 4), whereas some species, including the African *G. letus*, have setation throughout the orbital furrow (Fig. 9).

### Molecular analysis

**Figure 11.**
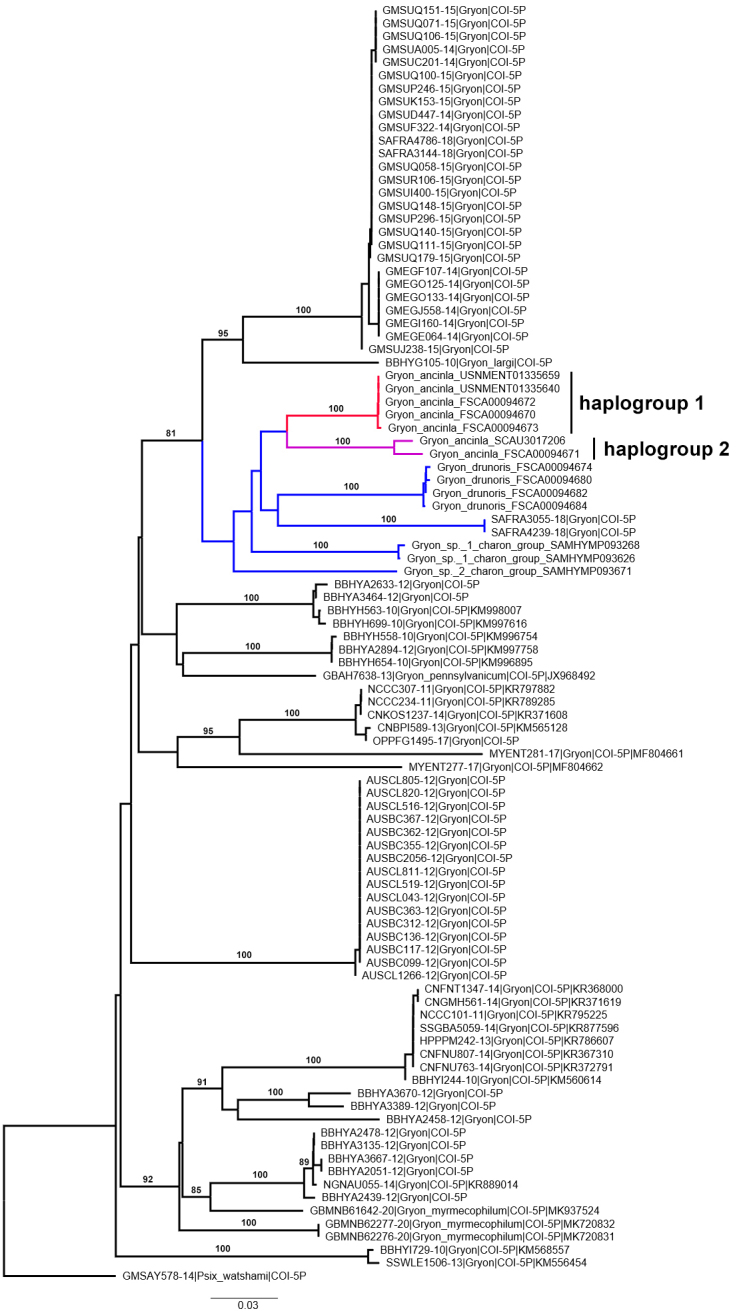
K2P neighbor-joining tree demonstrating the clustering of *Gryon* CO1 barcodes. The larger cluster in blue highlights the *Gryon charon* species group. Clusters in red and magenta highlight the *Gryon ancinla* haplogroups. Bootstraps values of 80 and above are indicated.

The neighbor-joining analysis revealed relatively large sequence divergences between clusters of *Gryon* CO1 barcodes (Fig. 11). Interpretation of sequence divergence in *Gryon* is currently hampered by the lack of species-level identifications that are necessary to define intra- and interspecific variation. We included 14 new CO1 barcodes from members of the *Gryon charon* species group and our neighbor-joining analysis recovered a cluster of these species with an additional unidentified *Gryon* from South Africa (BIN: BOLD:ADO2077; SAFRA3055-18, SAFRA4239-18) (Ratnasingham and Hebert 2013). We found two haplogroups of *G. ancinla*, indicated in Fig. 11 by the red and magenta branches. Specimens from each lineage were collected in a single Malaise trap sample in Guangzhou (FSCA 00094670–00094673), demonstrating that the haplogroups are sympatric. These *Gryon ancinla* haplogroups differ by K2P distances ranging from 9.6–10.4% (Table 1) and they are each other’s nearest neighbor. BLASTn searches yielded poor matches to the *G. ancinla* barcodes (86–87% identity to other hymenopteran barcodes). In BOLD, *G. ancinla* from haplogroup 1 were a 97% match to an unidentified specimen from Bangladesh (GMBCB2151-15) and the available image of this specimen is consistent with our concept of *G. ancinla*. This suggests that *G. ancinla* has a wide geographical distribution in southeast Asia.

## Discussion

*Gryon* contains widespread species and geographically broad analysis is needed to identify synonyms. This study characterizes a species found in southeast Asia to facilitate comparison with similar species of *Gryon* in the region and to associate ecological data with a taxonomic name.

## Supplementary Material

XML Treatment for Gryon ancinla

## Figures and Tables

**Figure 2a.**
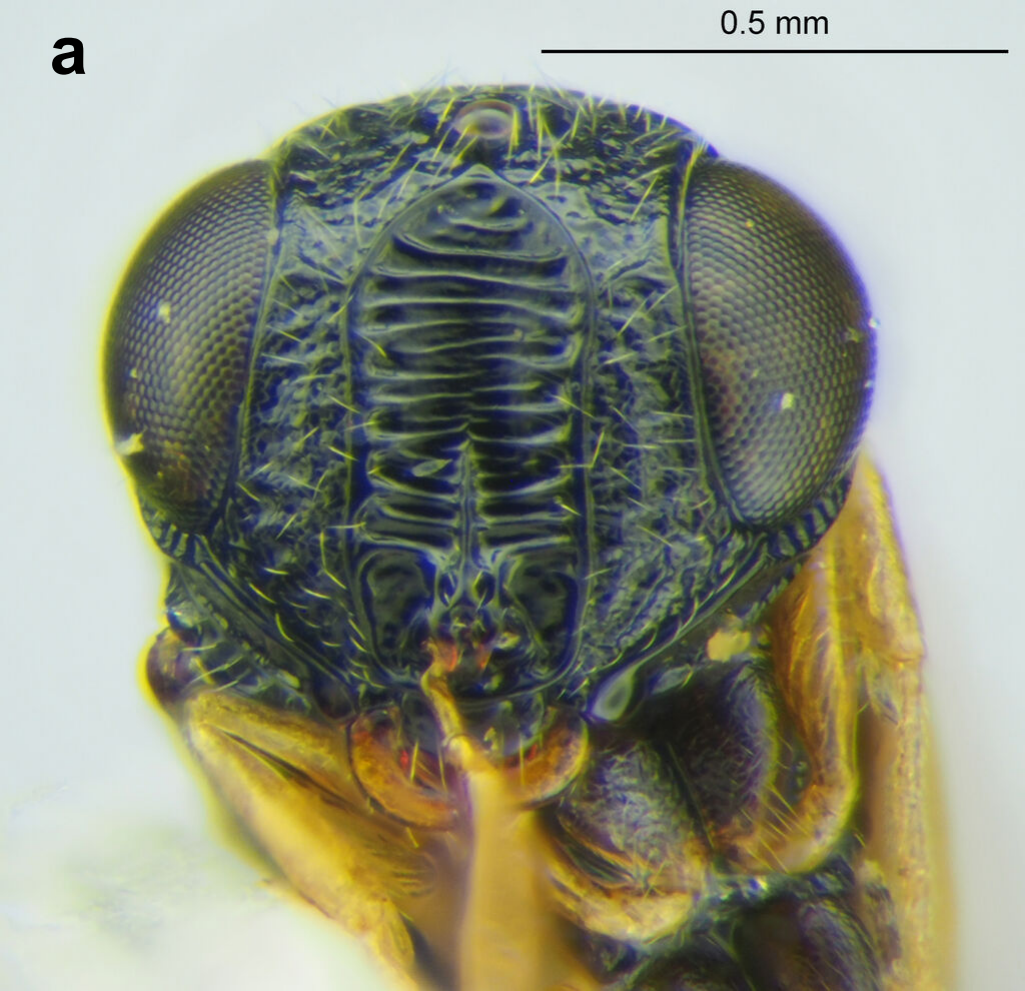
head, anterior view

**Figure 2b.**
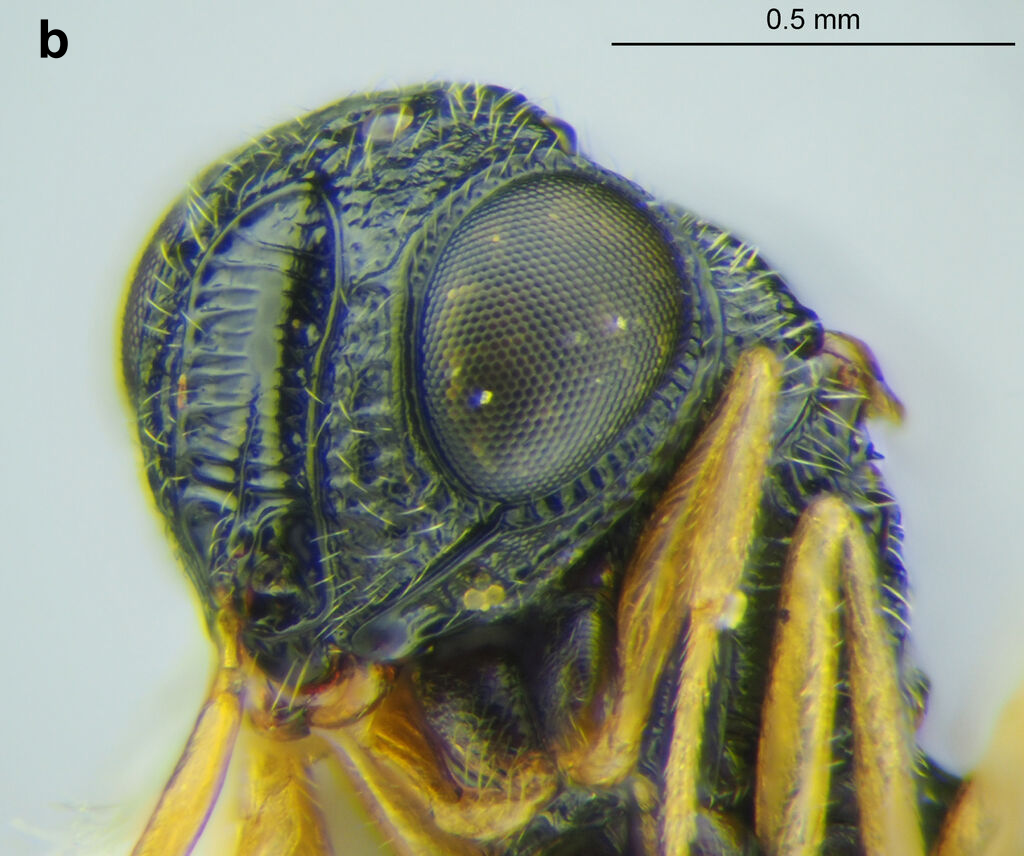
head and mesosoma, anterolateral view

**Figure 2c.**
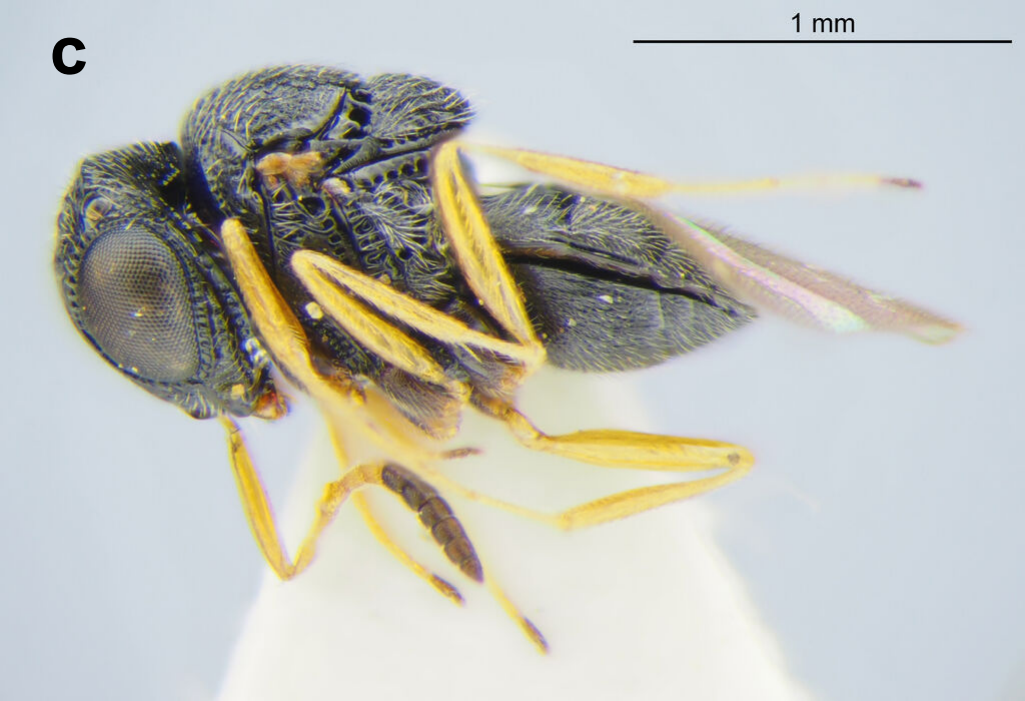
habitus, lateral view

**Figure 2d.**
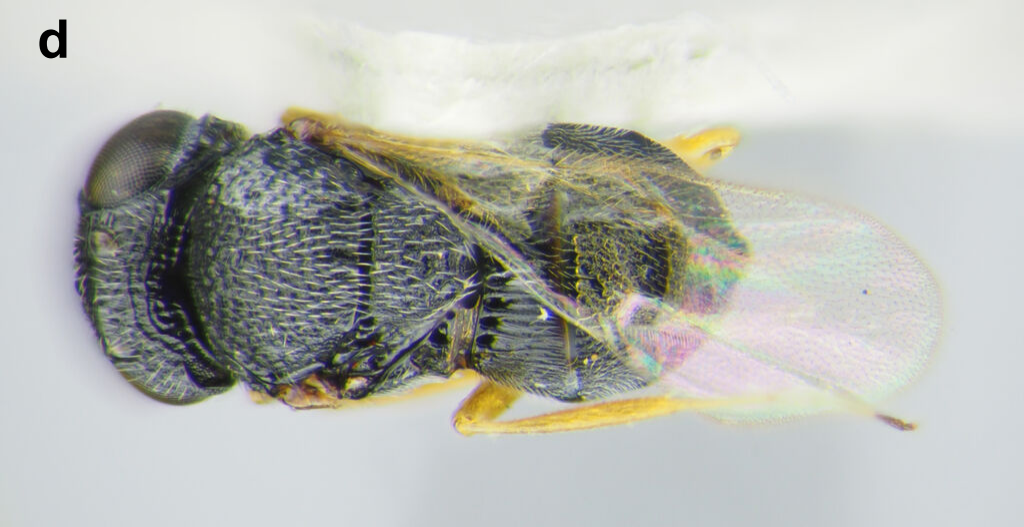
habitus, dorsal view

**Figure 3a.**
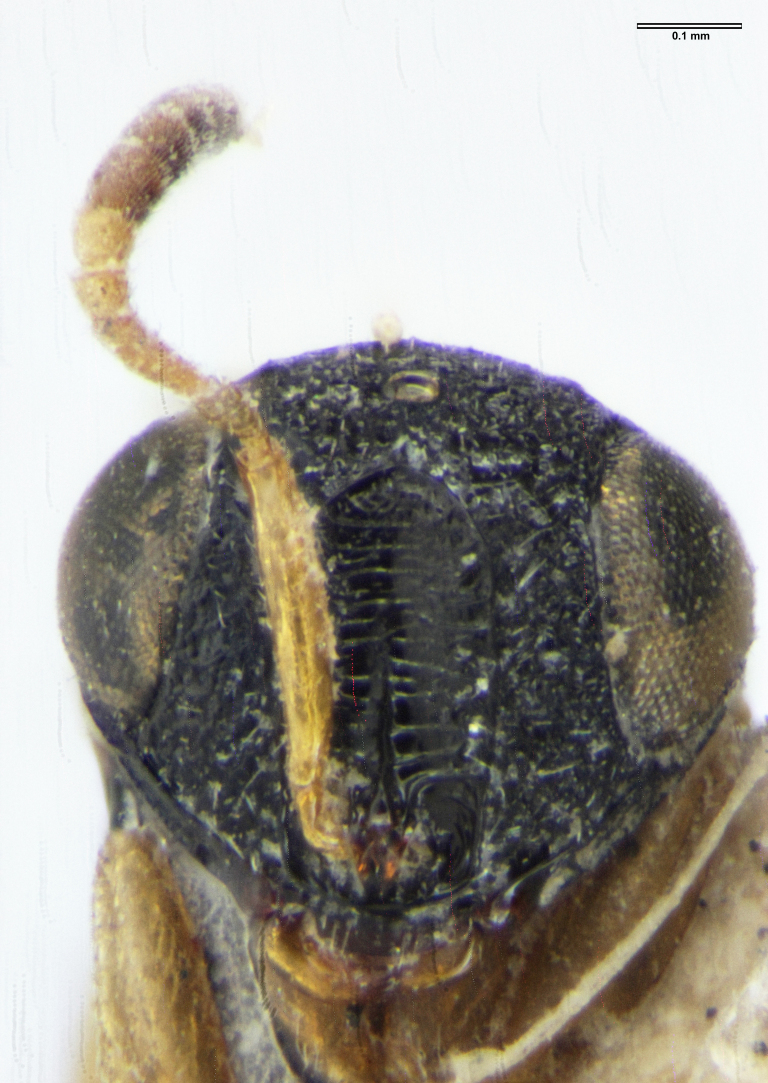
head, anterior view

**Figure 3b.**
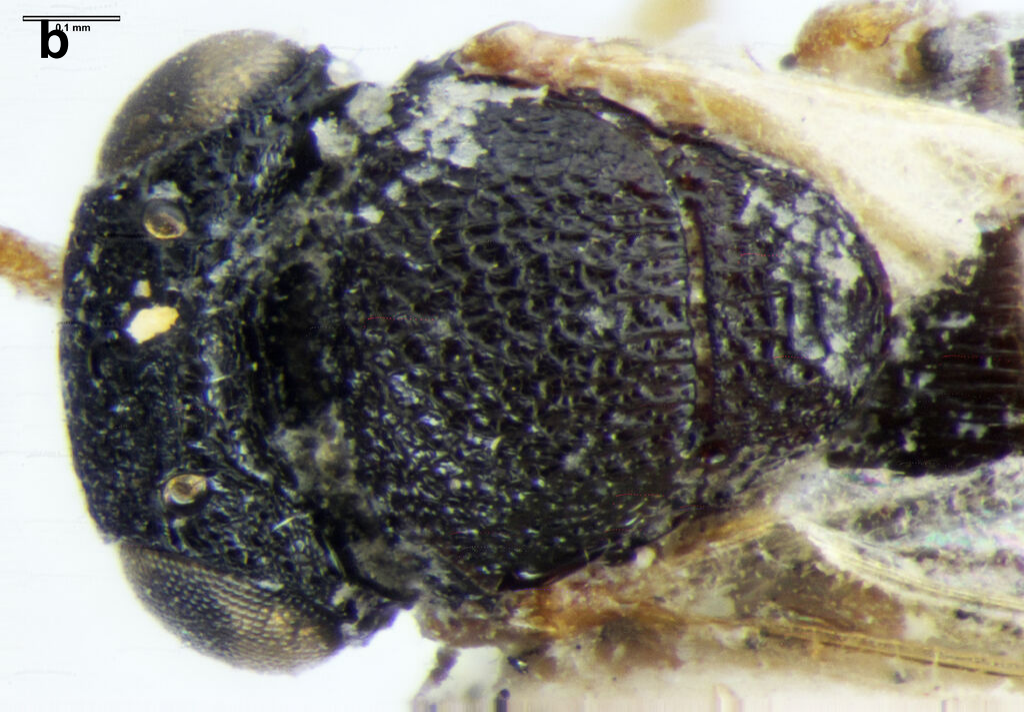
head and mesosoma, dorsal view

**Figure 3c.**
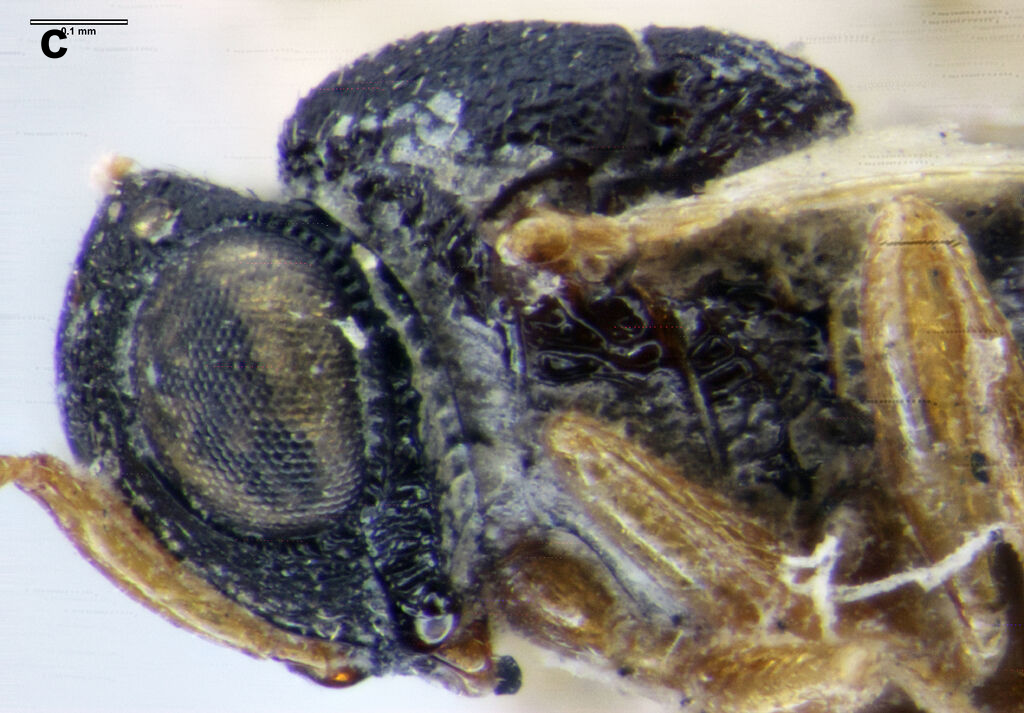
head and mesosoma, lateral view, image flipped horizontally.

**Figure 3d.**
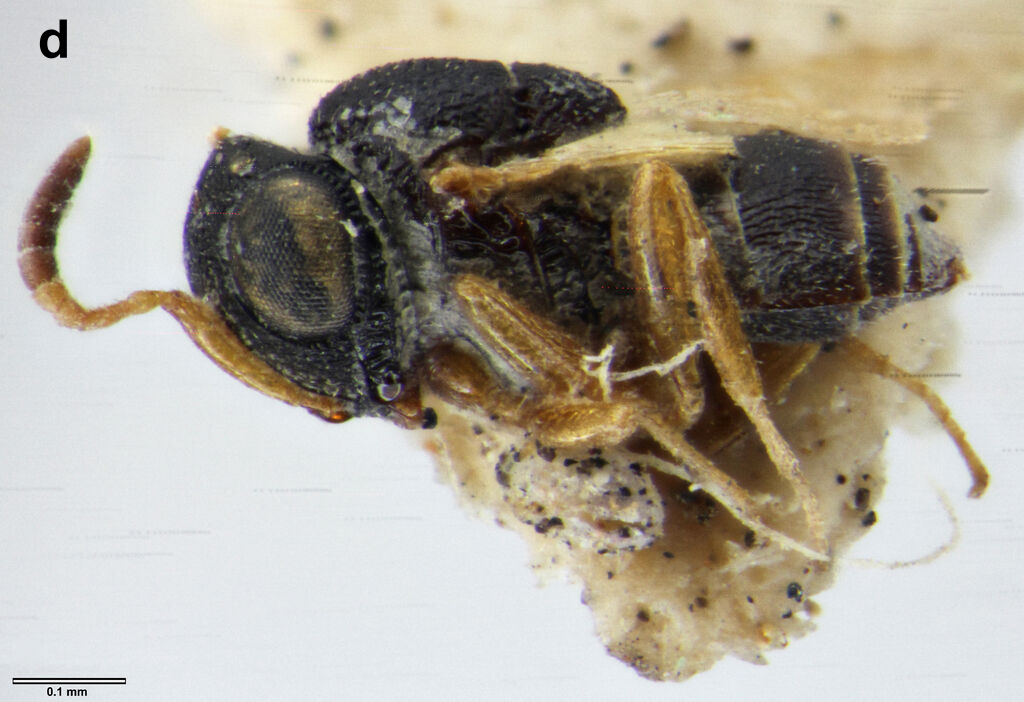
habitus, lateral view, image flipped horizontally

**Figure 5a.**
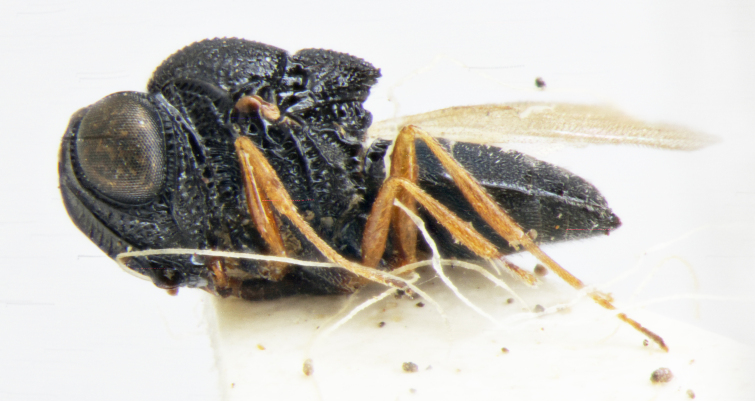
habitus, lateral view

**Figure 5b.**
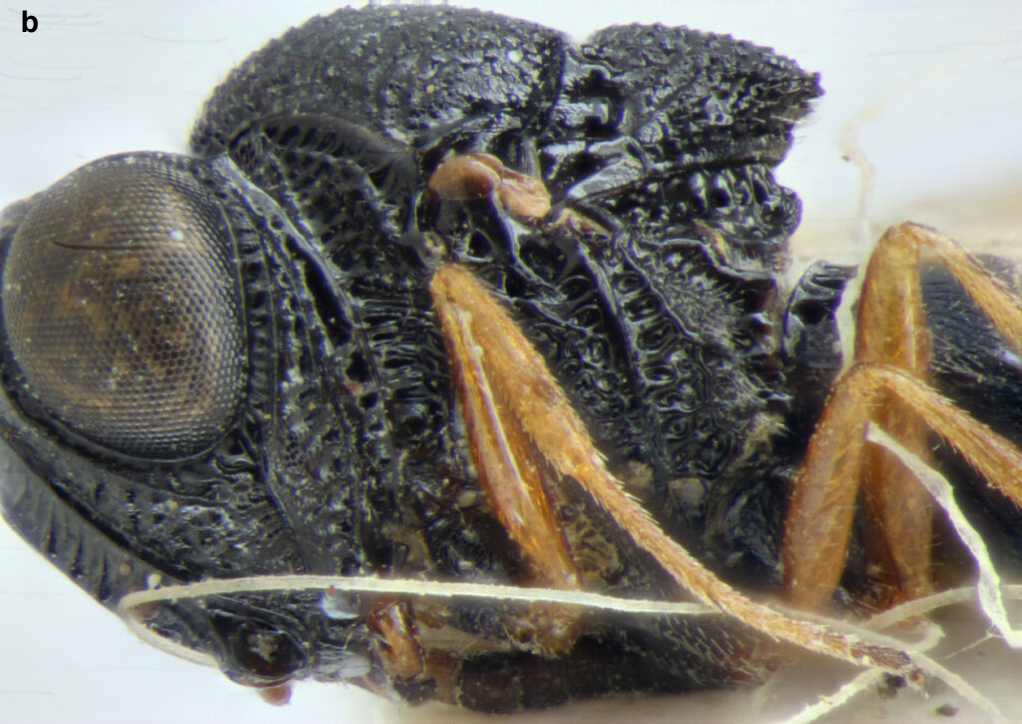
head and mesosoma, lateral view

**Figure 6a.**
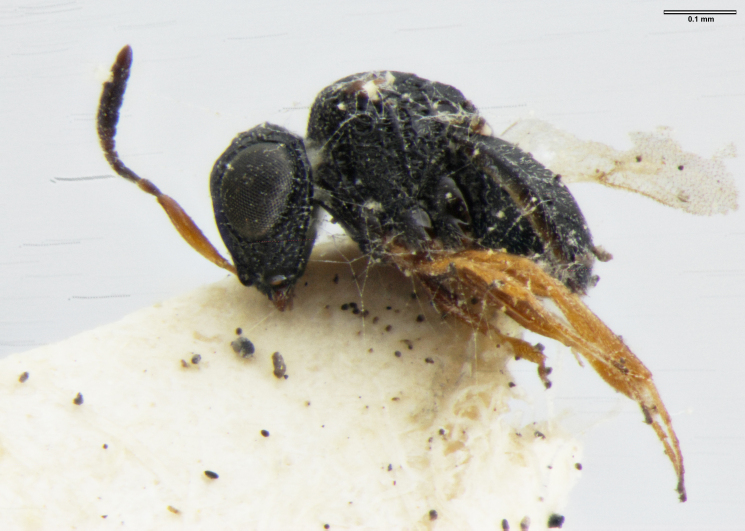
holotype female (IEBR 0176), habitus, lateral view

**Figure 6b.**
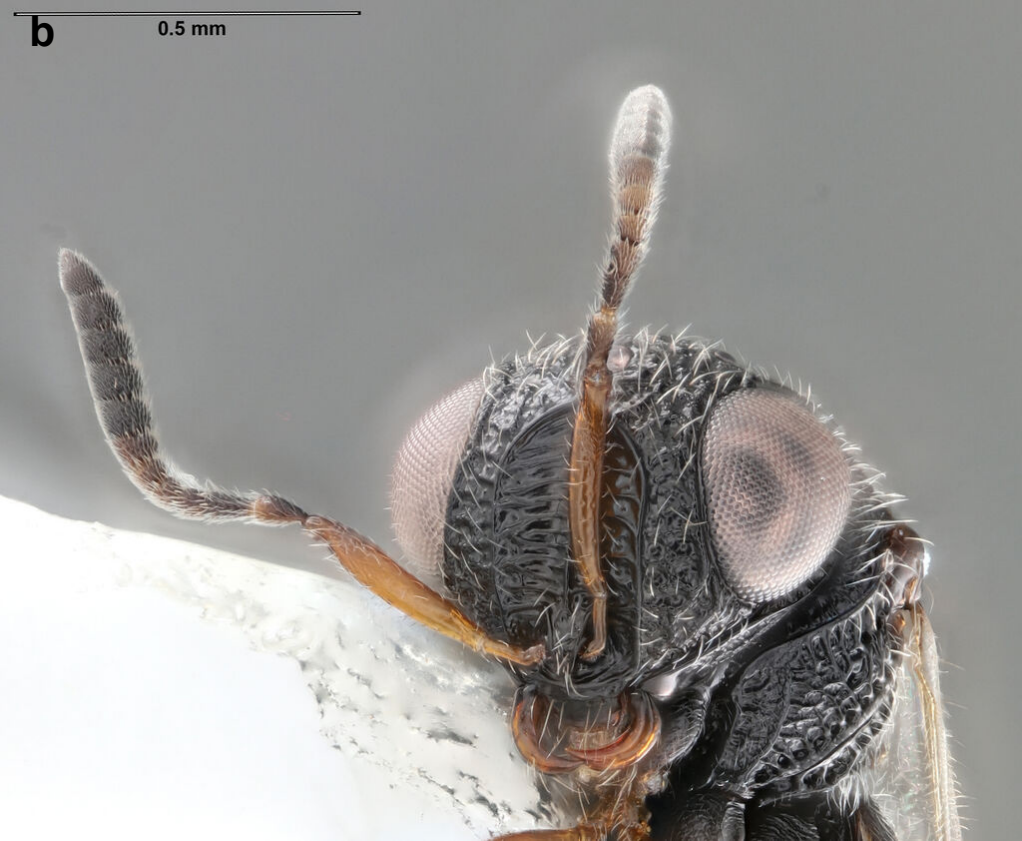
female (FSCA 00094680), head and mesosoma, anterolateral view

**Figure 7a.**
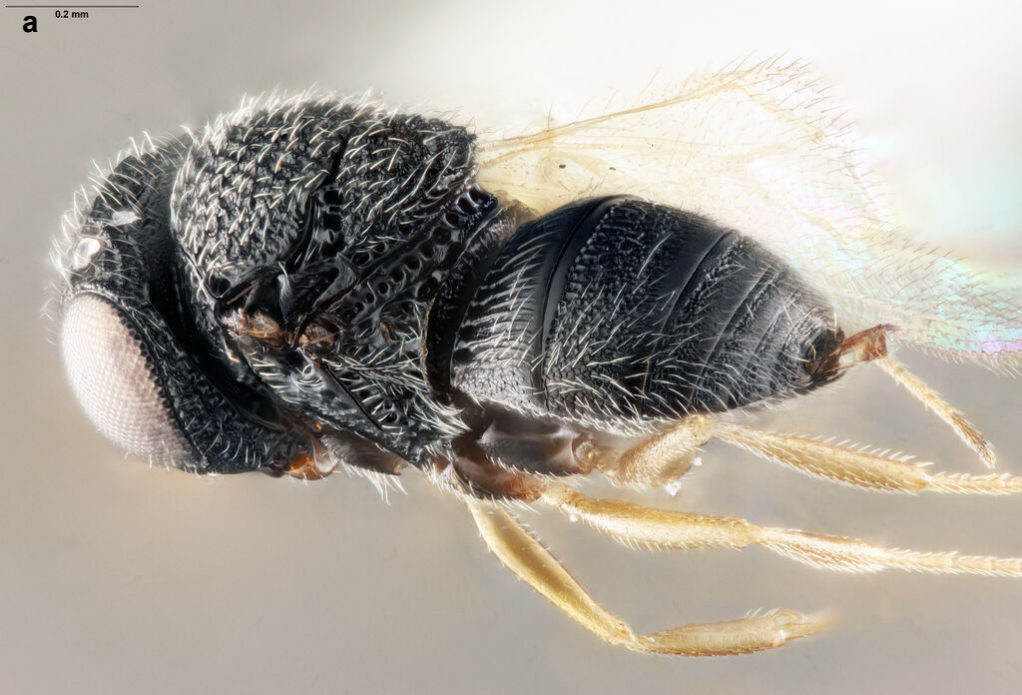
*Gryon ancinla* (FSCA 00090590), female, dorsolateral view

**Figure 7b.**
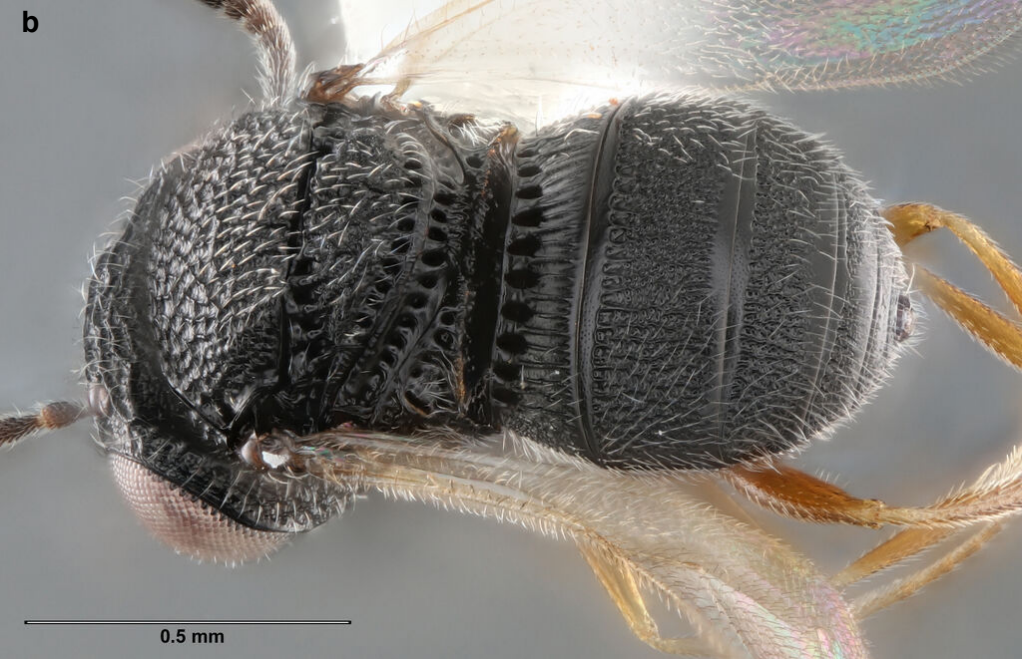
*Gryon drunoris* (FSCA 00094680), female, dorsal view

**Figure 8a.**
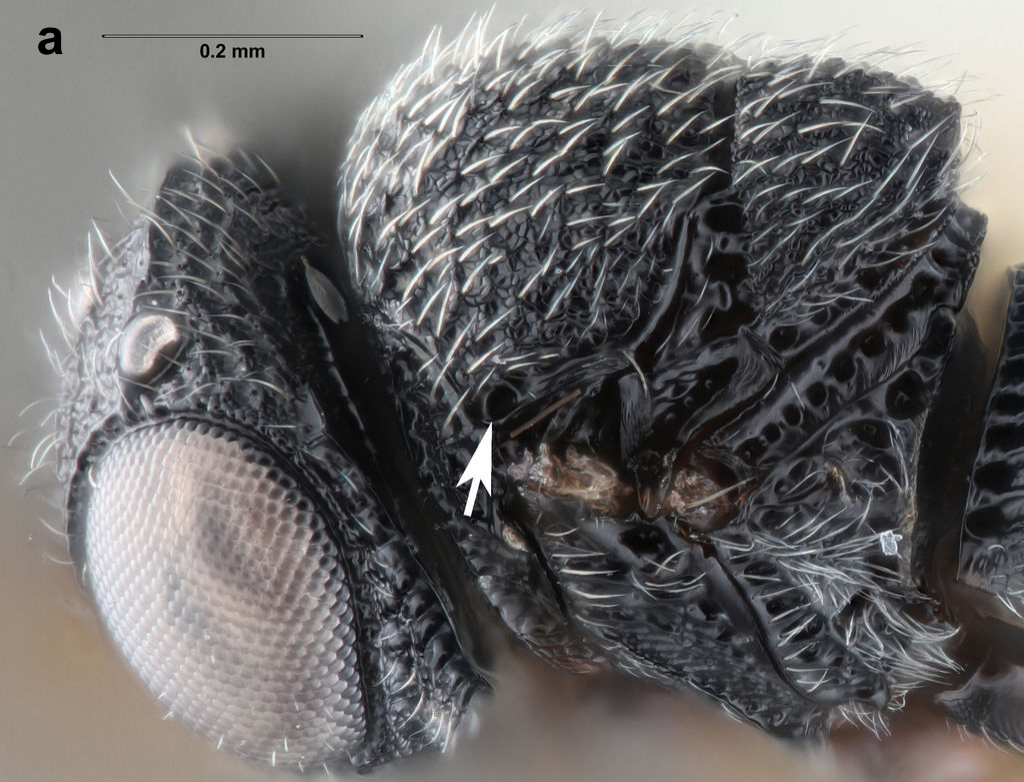
female (FSCA 00090590), head and mesosoma, dorsolateral view

**Figure 8b.**
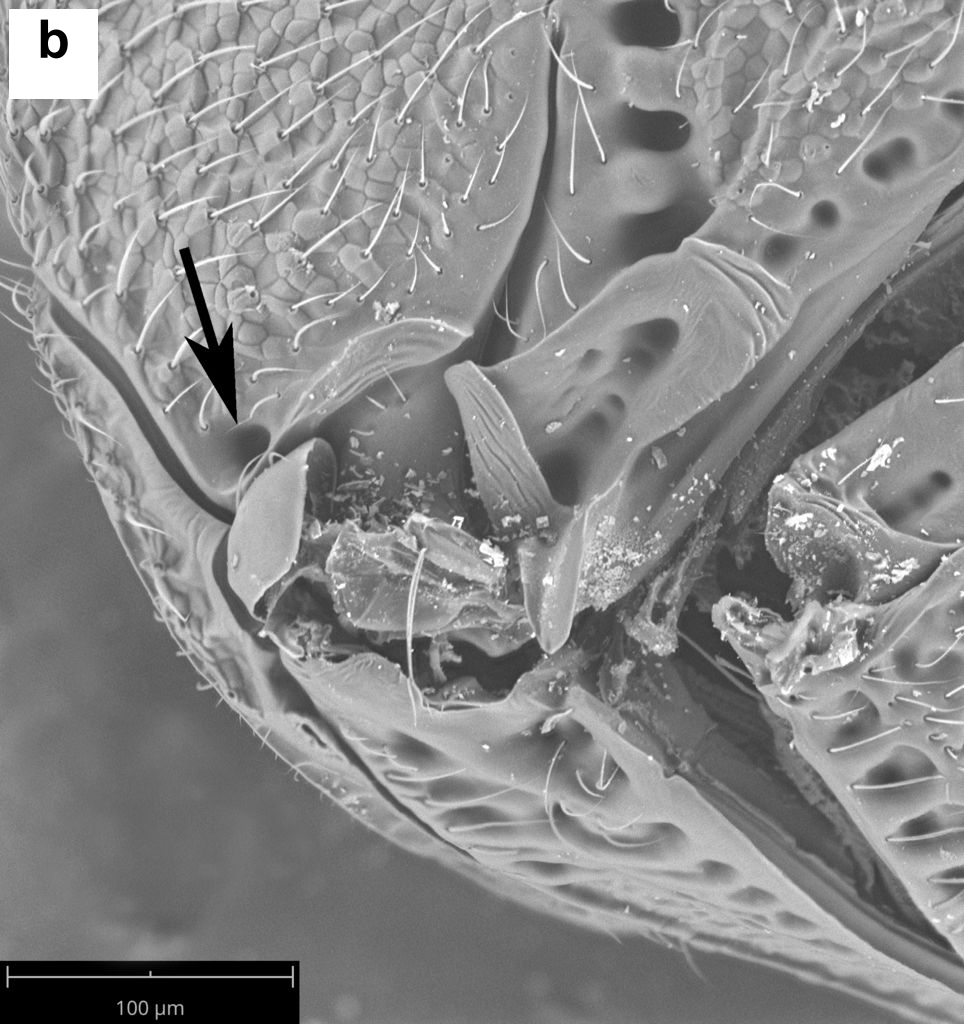
female (SCAU 3040175), mesosoma, dorsolateral view

**Figure 9a.**
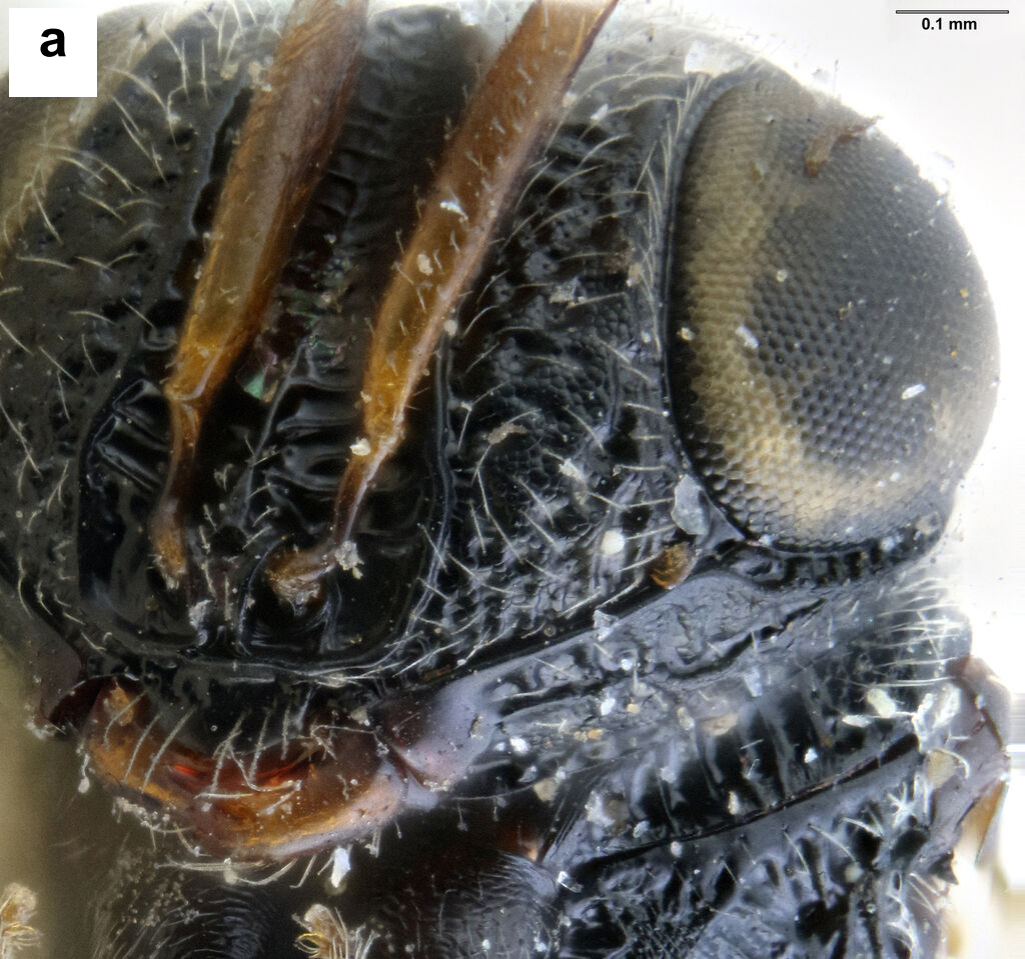
head, anteroventral view, image flipped horizontally

**Figure 9b.**
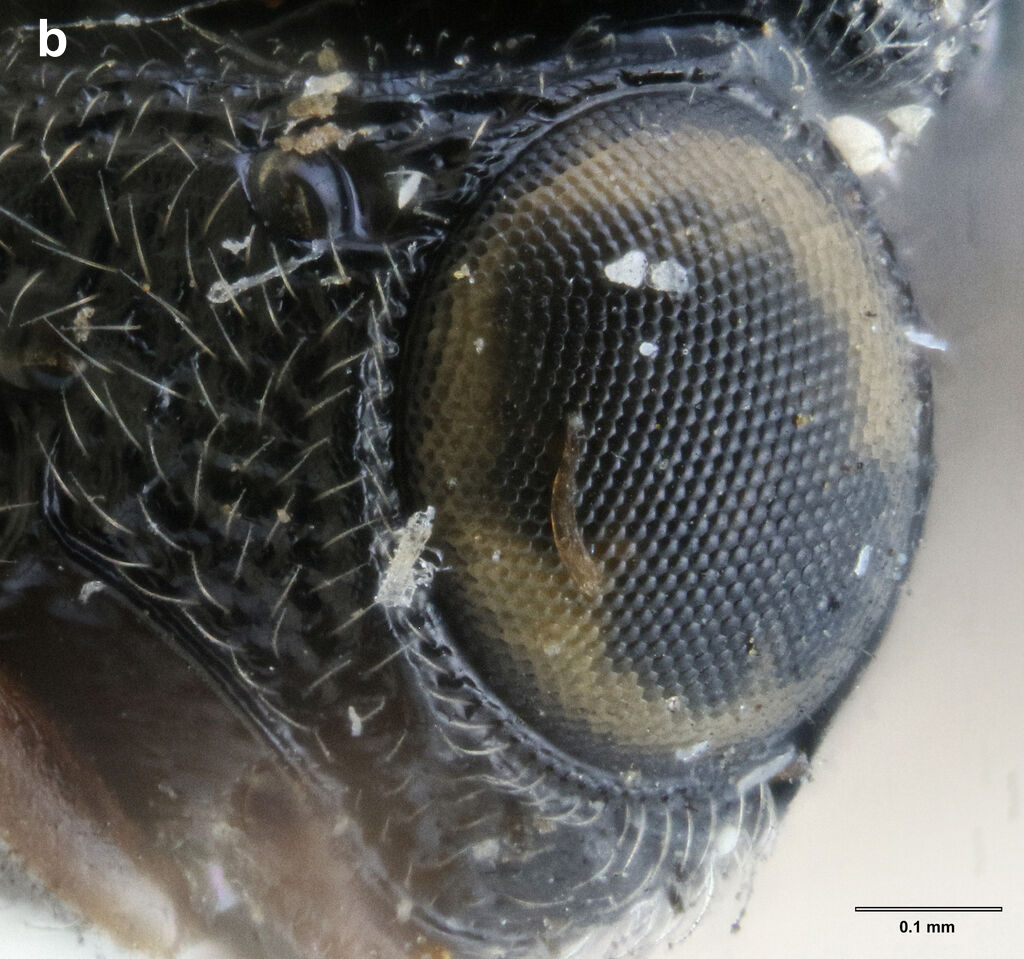
head, dorsolateral view, image flipped horizontally

**Figure 10a.**
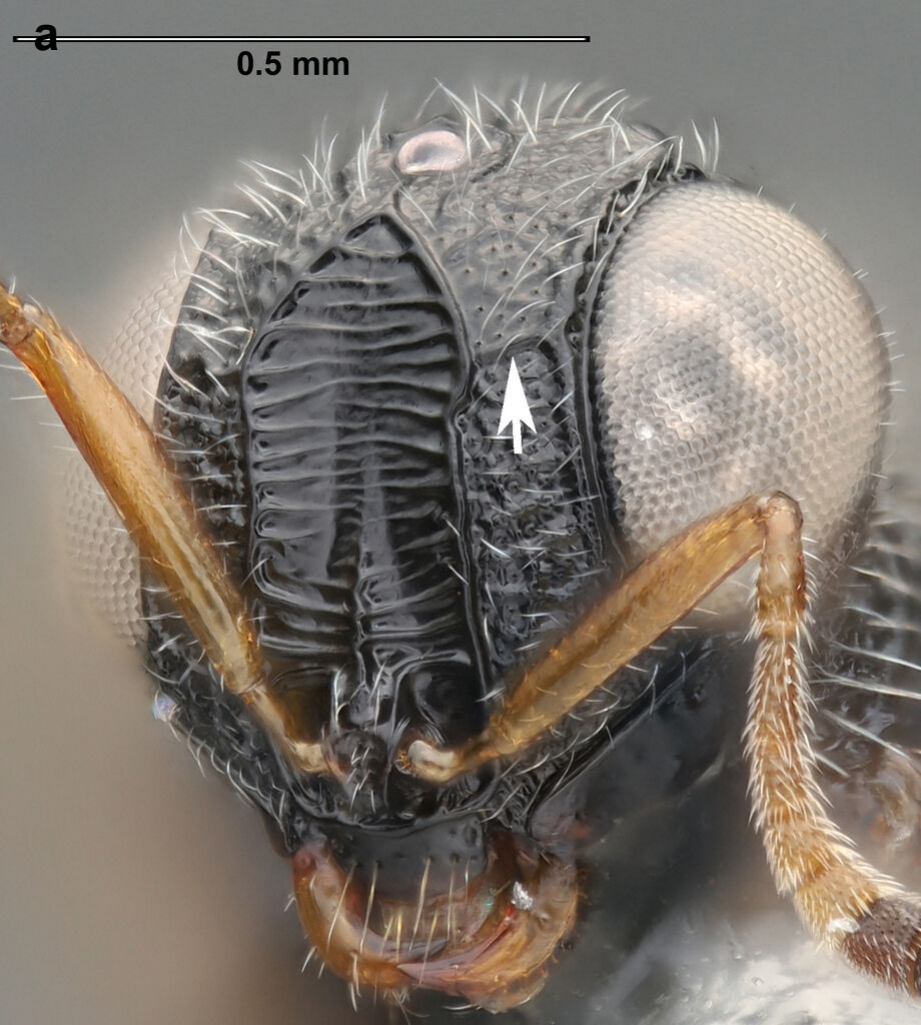
*Gryon ancinla*, female (FSCA 00094670), head, anterolateral view, image flipped horizontally

**Figure 10b.**
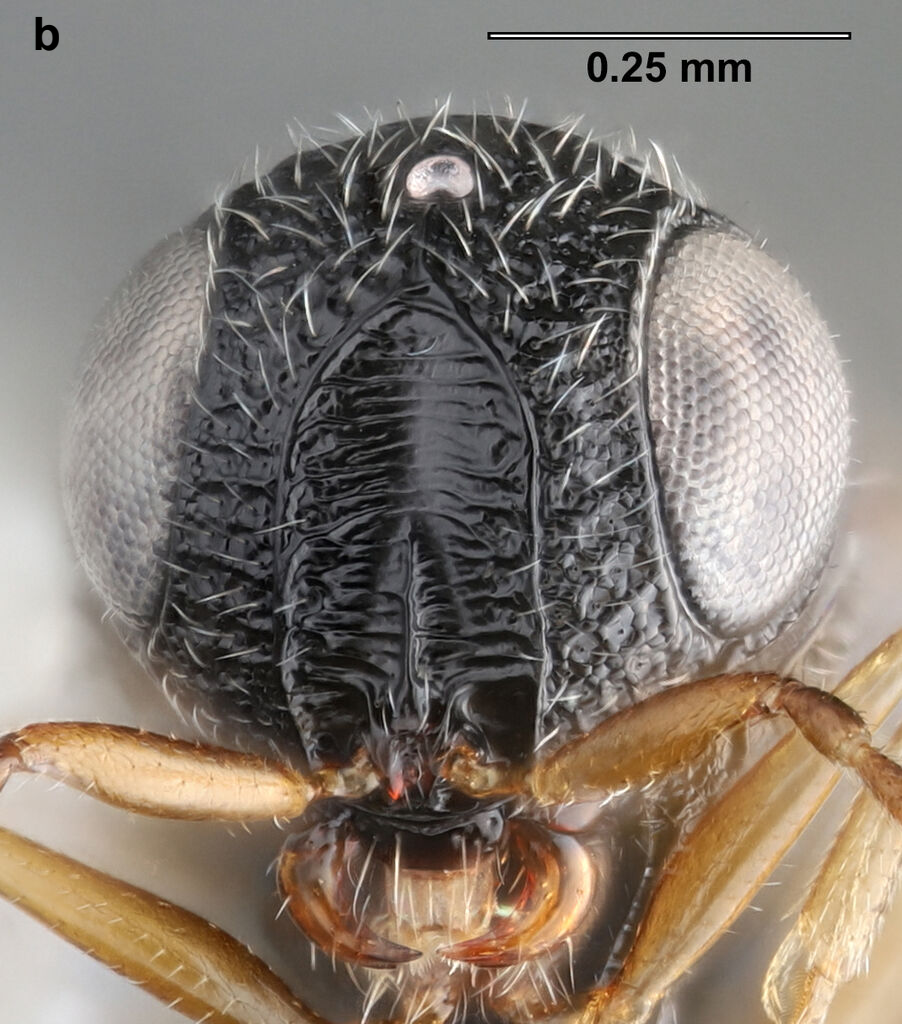
*Gryon ancinla*, female (FSCA 00094672), head, anterior view

**Table 1.** CO1 barcode K2P distances among species in the *Gryon charon* group.

***Gryon* Species Comparison**	**Range of Interspecific K2P Distances (%)**
*G. ancinla-G*. *drunoris*	12.9–14.8
*G. ancinla*-*G.* sp. (BOLD:ADO2077)	14.8–16.7
*G. ancinla*-*G.* sp. 1 *charon* group	11.4–13.7
*G. ancinla*-*G.* sp. 2 *charon* group	14.0–14.6
*G. drunoris*-*G.* sp. (BOLD:ADO2077)	15.7–16.1
*G. drunoris*-*G.* sp. 1 *charon* group	14.6–14.8
*G. drunoris*-*G.* sp. 2 *charon* group	16.2–16.6
*G.* sp. 1 *charon* group-*G.* sp. (BOLD:ADO2077)	16.4–16.6
*G.* sp. 1 *charon* group-*G.* sp. 2 *charon* group	15.0
*G.* sp. 2 *charon* group-*G.* sp. (BOLD:ADO2077)	16.2

**Table 2.** Specimens used in the molecular analysis

Species	Origin	Genbank Accession	Collecting Unit Identifier	Specimen Depository	DNA Depository
*Gryon ancinla*	China	MT604053	FSCA 00094670	FSCA	FSCA
*Gryon ancinla*	China	MT604054	FSCA 00094672	FSCA	FSCA
*Gryon ancinla*	China	MT604055	FSCA 00094673	FSCA	FSCA
*Gryon ancinla*	China	MT604056	USNMENT01335659	FSCA	EBCL
*Gryon ancinla*	China	MT604057	USNMENT01335640	FSCA	EBCL
*Gryon ancinla*	China	MT604058	SCAU 3017206	SYSU	SYSU
*Gryon ancinla*	China	MT604059	FSCA 00094671	FSCA	FSCA
*Gryon drunoris*	Vietnam	MT604060	FSCA 00094674	FSCA	FSCA
*Gryon drunoris*	Vietnam	MT604061	FSCA 00094680	FSCA	FSCA
*Gryon drunoris*	Vietnam	MT604062	FSCA 00094682	FSCA	FSCA
*Gryon drunoris*	Vietnam	MT604063	FSCA 00094684	FSCA	FSCA
*Gryon* sp. 1, *charon* group	South Africa	MT604064	SAM-HYM-P093268	SAMC	FSCA
*Gryon* sp. 1, *charon* group	South Africa	MT604065	SAM-HYM-P093626	SAMC	FSCA
*Gryon* sp. 2, *charon* group	South Africa	MT604066	SAM-HYM-P093671	SAMC	FSCA

**Table 3.** Species of *Gryon* in which the frontal depression is surrounded by carinae.

**Species**	**Basis of determination**
*G. ancinla* Kozlov & Lê	Examination of holotype
*G. charon* (Nixon)	Examination of holotype
*G. drunoris* Kozlov & Lê	Examination of holotype
*G. dasyni* (Nixon)	Nixon 1934
*G. hakonense* (Ashmead)	Examination of holotype
*G. ingens* Veenakumari & Rajmohana	Kamalanathan et al. 2016
*G. kenyotum* Mineo	Mineo 1982
*G. krishnagiriense* Sharma	Examination of holotype
*G. letus* (Nixon)	Examination of holotype
*G. lucmon* Mineo	Examination of holotype
*G. mudugeriense* Sharma	Examination of holotype
*G. nigriclavatum* (Dodd)	Examination of holotype
*G. odontogonusi* (Risbec)	Examination of lectotype
*G. oophagum* (Nixon)	Nixon 1934
*G. paracharontis* Mineo	Examination of holotype
*G. parakenyotum* Mineo	Examination of holotype
*G. philippinense* (Ashmead)	Examination of lectotype
*G. sponus* Kozlov & Lê	Examination of holotype
*G. urum* Mineo	Mineo 1982
